# Sustainable application of calcium carbide residue as a filler for 3D printing materials

**DOI:** 10.1038/s41598-023-31075-z

**Published:** 2023-03-17

**Authors:** Dmitriy E. Samoylenko, Konstantin S. Rodygin, Valentine P. Ananikov

**Affiliations:** 1 Institute of Chemistry, Saint Petersburg State University, Universitetsky prospect 26, Saint Petersburg, Russia 198504; 2 N. D. Zelinsky Institute of Organic Chemistry, Russian Academy of Sciences, Leninsky pr. 47, Moscow, Russia 119991

**Keywords:** Composites, Chemical engineering, Pollution remediation

## Abstract

Industrial activity results in ton-scale production of calcium carbide and generation of a significant amount of calcium carbide residue (CCR), which is often disposed of in the environment as waste. CCR is an active chemical, and rain washes away alkali from sludge, changing the pH of soils and water and damaging the environment. In this work, we explored new opportunities for the utilization of CCR in view of the coming industrial uptake of digital design and additive technologies. Amazingly, CCR can be successfully used as a filler for the modification of 3D printed materials towards the introduction of hybrid organic/inorganic frameworks. A series of commercially available plastics (PLA, ABS, Nylon, PETG, SBS) were successfully used as matrices for CCR-based composite production with high CCR contents up to 28%. Tensile analyses showed increases in tensile strength and Young’s modulus of 9% and 60%, respectively. Moreover, in comparison with the pure plastics, the CCR-based materials better maintained the digitally designed shape (lower shrinkage). Importantly, CCR-filled materials are 3D printable, making them very promising components in the building sector. Considering the amount of already available CCR stored in the environment, this material is available in large quantities in the near future for hybrid materials, and anticipated opportunities exist in the additive manufacturing sector. The involvement of CCR in practical composite materials is equally important for environmental protection and reuse of already available multiple-ton wastes.

## Introduction

**Figure 1:**
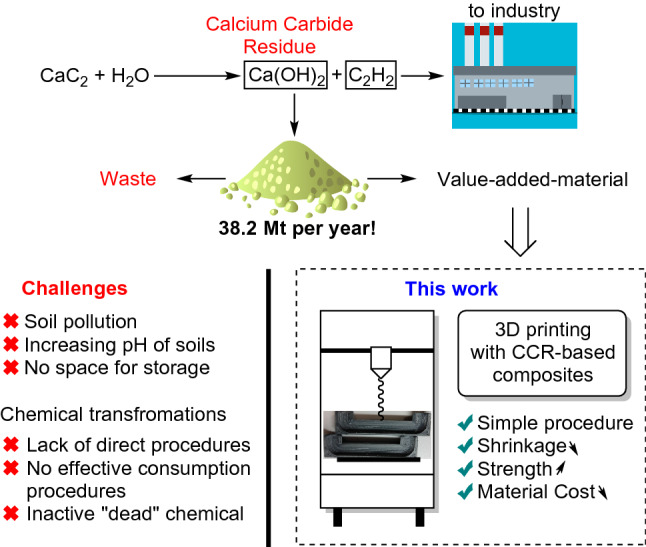
Production of acetylene from calcium carbide, accumulation of CCR, negative impact on nature and consumption in additive technologies studied in this work.

Modern industry needs acetylene as a key component for materials and chemicals production^1–4^. The scope of acetylene-derived products is very large: vinyl chloride^5–7^, acrylic acid^8–10^, butyndiol^11^, acetaldehyde^12–14^, and many others^15–21^. Manufacturing acetylene from calcium carbide is the current hydrocarbon-free method of industrial acetylene production. Moreover, calcium carbide has some advanced applications in organic synthesis^22–30^, catalysis^31–34^, mechanosynthesis^31,35^ and materials science^36^, with a continuous increase in interest in acetylene-based technologies due to the efficiency of atom-economic addition reactions. However, acetylene release is accompanied by the formation of calcium carbide residue (CCR), which is simply disposed and accumulates in landfills^37,38^. The accumulated CCR under the influence of rains can easily be washed away into soils, increasing alkalinity. There are a series of recent studies on carbide sludge utilization^39^ in the chemical industry^40–43^ and inorganic synthesis^44,45^. Other fields of CCR utilization involve CO_2_ capture^46,47^, soil stabilization^48,49^, materials^50,51^ and road construction^52^. Despite the efforts taken, it should be noted that the amount of generated carbide slag is rather large, and chemistry, as a standalone field, is not able to completely consume the generated CCR (Fig. 1)^53^. Therefore, there is an already realized risk for CCR to accumulate as waste without a long-term utilization strategy.

The construction and building sector may be a good opportunity for CCR utilization due to the significant consumption of materials circulating in this area (Fig. 1)^54–57^. However, raw CCRs are not applicable in construction due to their reactivity^58^, strong basicity and low plasticity. Moreover, a tendency to consume carbon dioxide from air^59^ requires immediate use of CCR after isolation, blocking the possibility of storing CCR without chemical modification for a long time^60^. Various additives may be added to stabilize CCR in the resulting mixtures, preventing the interaction of calcium hydroxide and carbon dioxide. Industrially available organic polymers can be used as a matrix to prevent possible interaction of CCR with external reagents.

Various inorganic particles and fibers are usually used to improve the strength of polymeric materials. The wide use of CCR as a filler in the development of new building materials with excellent strength properties makes it a promising filler in polymer composite materials as well. For this reason, we propose that CCR can be used as an easily available filler to prepare a composite material with an organic/inorganic hybrid structure. In this work, we address this topic and test the idea of compatibility of CCR with thermoplastics and applicability of the resulting composite material with additive technologies (Fig. 1).

The key question is whether the plasticity of the resulting CCR/plastic composites would be high enough to make filaments that are cheaper than pure plastics. New building blocks with advanced properties can be 3D printed, which is in demand in digitalized areas of construction, where flexibility and hybrid properties are demanded. The area of hybrid materials has developed rapidly with many actively explored opportunities^61–66^.

In the present study, the first examples of polymer composites were obtained from CCR and commercially available plastics: polylactic acid (PLA), polyethylene terephthalate (PETG), polyamide (Nylon), polyacrylonitrile butadiene styrene (ABS), polystyrene (HIPS), and polystyrene butadiene-styrene (SBS). The amount of CCR in the plastic varied in the range from 1 to 28%. The resulting mixtures were extruded to produce filaments fully suitable with commercially available 3D printing equipment. The filaments were charged in a 3D printer, and the application in 3D printing was rigorously tested, showing advantageous properties compared with pure plastics.

Description of novelty of the present study deserves a dedicated summary comment. Acetylene manufacturing from calcium carbide is accompanied by the generation of a significant amount of calcium carbide residue—a byproduct and waste that is simply disposed. The residue mainly consists of calcium hydroxide—an active alkaline compound that pollutes the environment. The processing of this waste is a challenging task due to its large amount and poor areas of application. The novelty of the present work is in the testing of residue as a filler to produce new composite materials to reduce the cost of commercially available plastics and to consume the carbide sludge.

## Materials and methods

### Materials

Calcium carbide was purchased from Sigma-Aldrich (purity > 75%, volumetric)^67^. Commercially available filaments, polylactic acid (PLA, Plastiq, 1.75 mm), polyethylene terephthalate (PETG, Bestfilament, 1.75 mm), polyamide (Nylon, Hi-Tech Plast, 1.75 mm), polyacrylonitrile butadiene styrene (ABS, Hi-Tech Plast, 1.75 mm), polystyrene (HIPS, Hi-Tech Plast, 1.75 mm), and polystyrene butadiene-styrene (SBS, Bestfilament, 1.75 mm), were used as received. Prior to printing, Nylon, Nylon-based composites and PETG were dried in an oven at 40 °C overnight.

### Characterization of the samples

The morphology of the CCR and paddles was studied on a Zeiss SUPRA 40VP scanning electron microscopy system. Good image quality was achieved using carbon deposition on the surface of the sample^68^ with equipment for vacuum deposition (Q-150T ES). The CCR particle size study was carried out using a Mastersizer 3000 laser particle size analyzer. The analysis was carried out at the refractive index of particles and the absorption index of particles—1.656 and 0.010, respectively. Water was used as a dispersant (refractive index 1.332). The study of mechanical properties was carried out using a Shimadzu AG-50kNXD testing machine. Mixing of CCR and polymers and hot extrusion for 3D printing were carried out using a filament extruder (Wellzoom). 3D models and G-codes were created using FreeCAD (v 0.19) and Maestro Wizard (v 3.6.0) software. FDM printing was performed on a Maestro Solo 3D printer.

### CCR preparation

CCR was obtained by hydrolysis of commercially available calcium carbide. After the reaction, the CCR was collected and dried to constant weight at 60 °C in an oven. The dried CCR was ground using an IKA A11 basic analytical mill and sieved through 100 µm sieves. Particles with an average diameter of no more than 100 µm were used for further investigations.

### Composite preparation

A commercially available polymer was crushed until the resulting pieces were 3–4 mm in length. An appropriate amount of CCR was added to the polymer, and both the matrix and filler were placed in the hopper of the extruder. Extrusion was carried out at maximum engine speed at 180–200 °C, depending on the nature of the polymer. The resulting material after cooling was reground to the original dimensions of the polymer (3–4 mm). The crushed precomposite material was extruded again using a nozzle with a hole diameter of 1.75 mm, and the extrusion temperature was 160–200 °C, depending on the material type.

### 3D printing of paddles

Paddles were created using a single additive manufacturing process. The diameter of the nozzle was 0.8 mm, and the layer height was 0.1 mm. The original 3D model was premodeled in FreeCAD. Then, using the slicer Maestro Wizard 3.6.0, the appropriate G-code was generated, which was not subjected to further editing. The generated G-code was used in subsequent 3D printing procedures. Before printing, the surface of the glass table of the 3D printer was cleaned of dust and treated with a commercially available adhesive (the 3D brand) to improve the adhesion of the part to the surface of the printed table. During printing, the temperature of the table varied in the range of 45–100 °C, and the temperature of the nozzle varied from 200 to 240 °C, depending on the material used. The print speed varied from 15 to 40 mm/s. More information is given in Table 1.

**Table 1:** 3D printing conditions.

Material	Temperature, °C		Cooling intensity, %	Extrusion multiplier, %	Printing speed, mm/s
	Build platform	Nozzle			
Nylon	100	230	0	100	30
Nylon–CCR	65	225	0	98	20
PETG	80	230	30	97	30
PETG–CCR	80	230	20	110	30
SBS	90	230	20	100	30
SBS–CCR	90	230	20	115	30
HIPS	100	230	0	100	30
HIPS–CCR	100	235	0	102	20
ABS	100	240	0	95	30
ABS–CCR	90	240	20	115	30
PLA	45	200	100	100	40
PLA–CCR	55	200	0	112	15

In all experiments, a nozzle with a hole diameter of 0.8 mm and a layer thickness of 0.1 mm was used.

## Results and discussion

In the present study, we explored the complete pathway, including generation of CCR, preparation of hybrid material, filament extrusion and testing in 3D printing. It should be noted that CCR is significantly different from pure calcium hydroxide^69^. The composition of CCR includes many impurities since the initial calcium carbide is of technical grade. Usually, black carbon, calcium carbonate, water, and some metal impurities contaminate CCR. Moreover, the main component of CCR, Ca(OH)_2_, can undergo significant changes during preparation. This is due to the reaction of Ca(OH)_2_ with CO_2_ from the atmosphere. The CaCO_3_ produced was confirmed by XRD and EDX (see Supporting Information). Note that the particle size of CCR also varies over a wide range. Thus, the use of CCR as a filler is a necessary condition. For practical reasons, it is important to follow a complete procedure with exact real components.

### Preparation of Nylon/CCR composites

**Figure 2:**
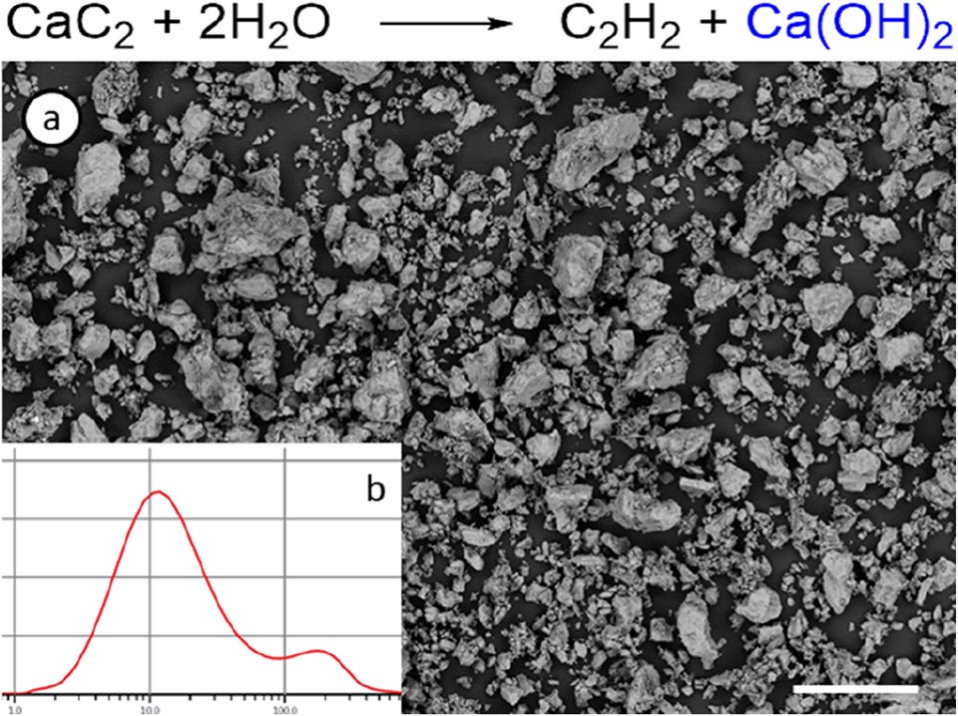
SEM image of CCR particles (**a**) and size distribution (**b**) after sifting (scale bar—100 µm).

Initially, CCR was isolated after the hydrolysis of calcium carbide (Fig. 2, top). The residue was collected and washed with water and then dried in an oven until constant weight. The shape, size, and morphology of the surface of CCR particles directly affect the final properties of the polymer composite material^70,71^. Therefore, particles with regular size and morphology were obtained using sifting sieves with a mesh size of 100 μm. Of course, particles with smaller diameters were also present in the final sample; however, larger particles did not pass through the sieves. A sequence of sieves can be used to select the required fraction and particles. Here, CCR with a particle size of approximately 100 μm was utilized (Fig. 2a). According to granulometric analysis of CCR particles (Fig. 2b), the average particle size was 66.7 µm, and the specific surface area was 501.4 m^2^/kg.

The potential of CCR as a filler was tested using a commercially available filament—Nylon (Table 2, entries 1–7). CCR powder was added to the ground Nylon wire, and the resulting mixture was extruded at 180 °C. After the first extrusion, an unstable mass was obtained, which was then ground again and extruded at the same temperature. After the second extrusion, a wire with a diameter of 1.6–1.8 mm was obtained. Furthermore, the resulting filament was used directly in 3D printing of samples for testing mechanical properties in tension. As a result, five samples of Nylon-based composite materials were obtained.

**Table 2:** Mechanical properties of the 3D printed plates based on Nylon composite.

Entry	CCR content, mas.%	Tensile strength, MPa	Young’s modulus, GPa	Elongation at break, %
1	0	39.59	1.12	253
2	1	26.75	0.94	134
3	3	33.16	1.10	255
4	5	38.17	1.19	111
5	10	36.31	1.35	83
6	20	43.14	1.79	110
7	28	25.70	1.12	44

According to tensile tests of the 3D printed samples, an increase in filler content resulted in an increase in the Young’s modulus and tensile strength and a decrease in the elongation at break (*cf*. literature data)^72^. The addition of 1–3 mas.% (Table 2, entries 2–3) of the CCR filler resulted in noticeable inclusion of agglomerated particles in the composite, which significantly decreased the relative elongation at break and the tensile strength due to the lower surface activity of the agglomerated particles^73^. When the mixture of filler and plastic is passed through the extruder, the initial heating of the plastic promotes the formation of a shell of filler that adheres to the surface. With further melting and mixing, the shell is deformed, which provides agglomeration under the influence of “hydrophilic-hydrophobic properties” or interaction with another such shell. With further heating, the plastic is softened and melts, reducing the viscosity of the system as a whole, and only the friction of the polymer matrix becomes insufficient to break large agglomerated particles. Moreover, due to the small amount of filler, individual agglomerates can move freely in the free volume of the polymer matrix without interacting with the environment. Agglomeration of CCR was also confirmed by SEM studies (Fig. 2a and Supporting Information). As a result, the adhesion of the polymer matrix to the filler was reduced, and the strength of the agglomerated particles was less than the strength of the separated particles, resulting in earlier rupture of the sample. The properties of the composite containing 3 mas.% filler were similar to those of pure Nylon (Table 2, entry 1). Interestingly, a further increase in the filler amount results in a fairly uniform distribution of particles without changing the preparation procedure. An increase in the CCR amount (Table 2, entries 4–6) led to an increase in the strength up to 20 mas.% filler (Nylon-CCR), when elongation of the sample (110%) at break was expected to decrease, and a significant increase in Young's modulus by 60% and tensile strength by 9% was noticed. A CCR content of 28% (Table 2, entry 7) provided a significant decrease in the mechanical test of the composite, limiting further filling. Thus, the best resistance properties were achieved with a filler content of 20 mas.%, and in further investigations, this composite was used. Note that optimization of the filler/matrix ratio provided valuable 3D-printable materials^74–77^.

### Testing other polymer matrices

Encouraged by the promising results in the utilization of CCR as a filler of Nylon-based materials, commercially available polymers were used to extrude new composites: PLA, PETG, ABS, HIPS, and SBS (Table 3, and S7 in Supporting Information).

**Table 3:** Mechanical properties of composites.

Parameter	ABS-CCR	PETG-CCR	HIPS-CCR	SBS-CCR	PLA-CCR^a^
Tensile strength, MPa	38.6 (49.0)^b^	24.6 (56.0)	24.2 (32.4)	20.4 (20.1)	– (64.3)
Young’s modulus, GPa	1.95 (1.62)	2.32 (1.70)	2.16 (1.93)	1.19 (0.98)	– (2.58)
Elongation at break, %	6.8 (17.3)	1.6 (9.7)	2.0 (5.6)	10.5 (17.2)	– (3.7)

The CCR content is 20% by weight in all cases. Similar to nylon-based composites, 5 samples of each material were printed for tensile tests.

^a^In the case of PLA, the introduction of CCR led to a significant increase in the fragility and inability to study properties.

^b^The values for the pure initial polymers are in parentheses.

To achieve a homogeneous distribution of the filler inside the polymer, the extrusion of the composite was carried out 2–3 times until the visible agglomerated particles disappeared at 180–190 °C (depending on the polymer). Moreover, in this way, we managed to establish the possibility of reusing the obtained composite materials. In the cases of PETG and PLA, fragility development of the resulting wire with a new extrusion cycle was observed. As a result, the mechanical properties of the printed product were changed (Table 3, PLA-CCR). However, the PETG-based material after the 2nd re-extrusion retains good properties for printing, while the PLA product crumbles in the hands. Nylon, SBS, ABS and HIPS have proven to be more resistant to repeated extrusion cycles, making them more promising for use. The brittleness of ABS, PETG, and HIPS polymers with 20% CCR varied due to decreasing tensile strength by 21%, 56% and 25%, respectively. However, the Young’s modulus increases by 21%, 36%, and 12%, which indicates an increase in the hardness of the composites. Despite the high elasticity of SBS and its resilient properties, the elongation at break was only 17.2% compared with pure Nylon (253%). In the case of pyre SBS, the polymer filled with 20% CCR resulted in an increase in tear resistance. The ultimate strength of the composite decreased by less than 1%, and Young's modulus increased by 21.5%. Thus, 20% CCR filler resulted in more brittle polymers, deteriorating mechanical tests; therefore, filling the polymer matrix is not suitable for all polymers with low plasticity. The use of polymers with high plasticity is favored compared to the use of harder polymers.

### 3D printing with the obtained composites

**Figure 3:**
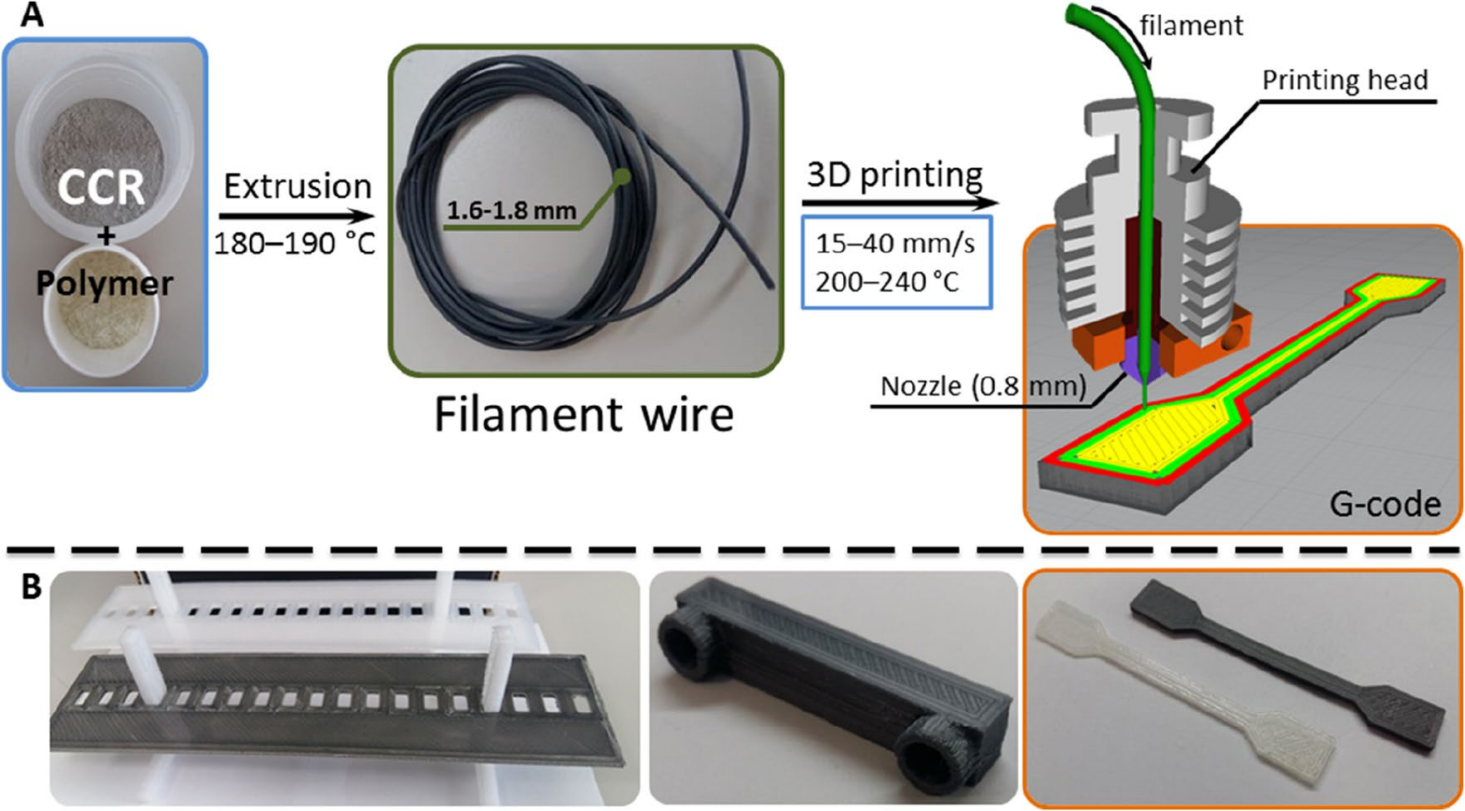
(**A**) Production and usage of CCR-based composite materials in 3D printing; (**B**) examples of 3D-printed objects produced from Nylon-CCR and initial Nylon.

Since the largest particles of CCR were no more than 200 µm (Fig. 2a, Supporting Information), a nozzle with a diameter of 0.8 mm was chosen to avoid the accumulation of CCR particles inside (Fig. 3A). Larger nozzles were not able to hold the filament due to the lower viscosity of CCR-based composites. Optimization of the 3D-printing conditions was carried out for each pure polymer and composite (see Table 1 in “Materials and methods” section and S2 in Supporting Information). After optimization, a series of objects was printed (Fig. 3B), and the corresponding samples were studied using a tensile testing machine and SEM (see Supporting Information). According to SEM, no expansions between the layers were detected. Both naked and covered polymeric matrix particles of CCR were detected on the surface of the composite material Nylon-CCR using EDX analysis (see Supporting Information). Pores were also detected on the surface, presumably due to water evaporation during extrusion. Interestingly, the Nylon-CCR composite was less hygroscopic (moisture content 0.96%) than pure Nylon (1.24%).

**Figure 4:**
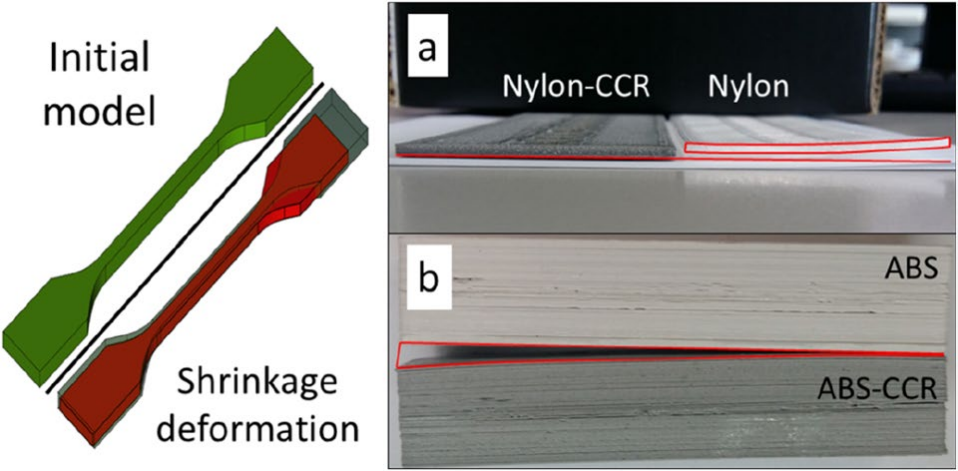
Two long shapes printed with pure Nylon and the Nylon-CCR composite (**a**) and the distortion of shapes made from pure ABS and the ABS-CCR composite, respectively (**b**).

Shrinkage is another important feature that affects the predictability of the properties and dimensions of final parts made from polymeric materials^78^. Shrinkage causes mechanical stress inside the material, and high values lead to faster destruction^79^. After printing, shrinkage can be estimated visually by comparing pure polymers and composites (Fig. 4). Obvious “twisting” of a paddle-shaped detail was detected in the case of pure Nylon, and the composite-based detail was not twisted (Fig. 4a).

The shrinkage value was calculated from the longest edge of the shape and compared with the corresponding value set in the 3D model.

$$S=\frac{{L}_{c}-{L}_{m}}{{L}_{c}}\times 100{\%}.$$

Here, S is the shrinkage value, %; L_c_—length of the object, according to the 3D model, mm; L_m_—actual length of the part, mm. Thus, for the Nylon material, a shrinkage value of 5% was observed. ABS-based objects are known to undergo shrinkage^80^. Interestingly, neat ABS does not shrink under standard conditions (Fig. 4b); however, the composite-based ABS-CCR shape was distorted.

Another test of print quality compared the practical dimensions of the resulting part with the theoretical dimensions of the 3D model (for more information, see Supporting Information, part S6). During the test, 3 identical parts with internal cutouts of different geometric shapes were printed in parallel. The analysis of linear regression functions showed that the part made of the selected composite material (SBS-CCR) respects the specified geometry in the same way as commercially available plastics (ABS, SBS). Thus, in the case of flexible polymer matrices, CCR reduced the shrinkage, and in the case of solid matrices, CCR increased the distortions. Shrinkage, strength, and the other parameters of hybrid materials depend on the internal organization of the polymer matrix and filler. To identify regularities in the properties and structure, we decided to study the microstructure of the composite materials.

### Microstructure investigations of the obtained hybrid materials

Controlling the microstructure of hybrid materials is an interesting aspect in studying and understanding the properties of filled materials. Both the final strength of the material and the mechanism of its destruction depend on the interaction of the polymer matrix and filler. The nature of such an interaction can vary from chemical bonding of components to the formation of hydrogen bonds and other nonvalence interactions.

Using SEM and EDX, we studied the obtained hybrid materials in order to identify differences in the structure of the obtained materials. Moreover, with the help of EDX, it was possible to fix the approximate elemental composition of the filler particles. In all cases, calcium is related to oxygen at 1:3 or 1:2, which additionally proves the formation of CaCO_3_ during the preparation of these composite materials. In addition, encapsulated calcium hydroxide is able to remain inside the polymer matrix in isolation from external influences.

**Figure 5:**
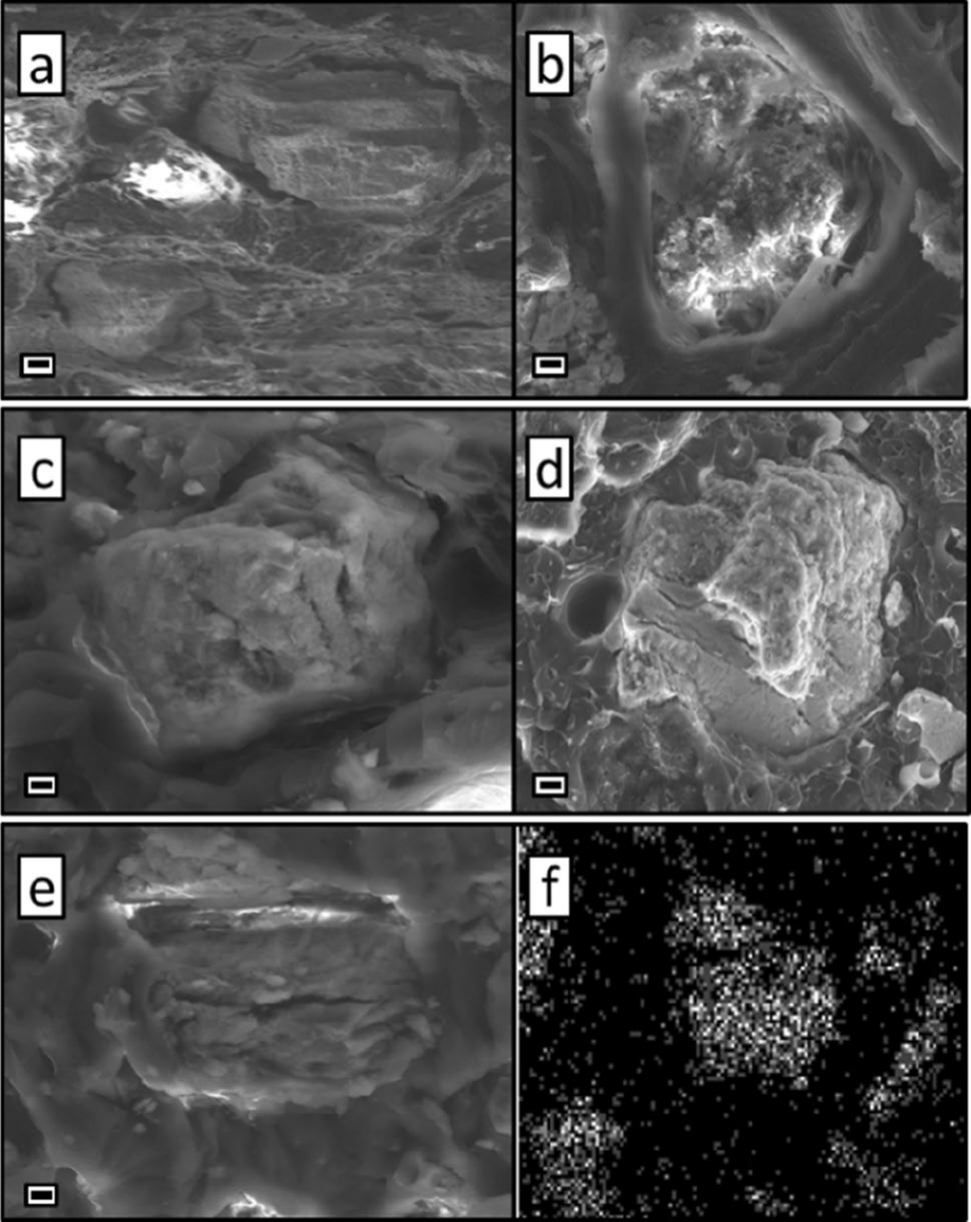
SEM investigation of the microstructural organization of (**a**) ABS-CCR, (**b**) SBS-CCR, (**c**) PETG-CCR, (**d**) HIPS-CCR, and (**e**) Nylon-CCR. (**f**) CRS map for the Ca element in the case of SBS-CCR material. Scale bar 2 µm.

Figure 5a shows the CCR particle distribution in the ABS plastic. In this case, a clear phase boundary between the filler and plastic was observed. This result may indicate the absence of a strong interaction between the polymer and the filler capable of holding the two phases together. In this regard, a possible mechanism for the destruction of such a material during tension is the expansion of existing voids around the filler with subsequent destruction, while the filler particles apparently could not slow down the propagation of the formed crack. This can be seen from the preservation of the integrity of the exposed particles on the cut. It is possible that the presence of these voids affected the decrease in the tensile strength index of this composite material.

In the case of using SBS (Fig. 5b, f), a different picture is observed. Note that the plastic itself, unlike ABS, has good elastic properties. Therefore, amorphous polymer fibers and threads are visible in the cut of the resulting composite. Interestingly, in the polymer-filler interface, similar threads can be seen moving away from the CCR solid particles, which indicates close contact and adhesion of the polymer matrix to the filler, due to which the material was additionally strengthened. Thus, when such a material is destroyed, the formation of destructive pores can be inhibited due to the work of adhesion between the polymer and CCR. This is also confirmed by experimental data, where Young’s modulus increases and elongation at break decreases. Moreover, it is noticeable in the photo that many of the exposed particles collapsed along with the polymer. This result once again shows the cooperative contribution of the matrix and filler to the strength properties of the material.

Plastic based on polyethylene terephthalate combines a good combination of hardness and elasticity. Hybrid materials based on it, on the contrary, are not distinguished by elasticity but have significant rigidity. The study of its microstructure (Fig. 5c) revealed that, as in the case of ABS plastic, most filler particles have a cavity around them that separates the filler and the polymer matrix. In isolated cases, partial adhesion of plastic to the filler is observed. According to the nature of the destruction, it can be said that the acquired rigidity of the material, together with the voids formed at the interface between the matrix and the filler, poorly prevent the propagation of destructive cracks. The filler particles have retained their integrity, so the main role in ensuring the strength properties of the material is played by the plastic used.

HIPS in 3D printing is mainly used to create support structures for overhanging parts of the main product. For this reason, superior performance properties are not initially required from this plastic since after printing, it is dissolved to separate from the product and disposed of. A slice of the HIPS-based composite (Fig. 5d) shows a picture similar to the PETG-based samples. At the plastic-filler interface, a partial interaction of the components is observed, which did not significantly improve the performance properties of the original plastic. However, tensile test results showed that HIPS-CCR and PETG-CCR have almost identical properties but are less brittle and more recyclable.

Nylon, similar to SBS, possesses good resilience (Fig. 5e). Products from this polymer have good strength, elasticity and resistance to abrasion. Composites based on it showed the best results in tensile tests. As in the case of SBS, deformed “fibers” of plastic and destroyed particles were found at the cut point. In addition, it should be noted that there are no voids between the polymer matrix and the filler, which indicates their interaction with each other. As expected, cavities are seen in areas with destroyed filler particles. Thus, the properties of the resulting material are provided by both the polymer matrix and the filler particles. Interestingly, the identity of the microstructure for Nylon-CCR and SBS-CCR did not help explain the prominent properties of HIPS-CCR. Comparing the nature of these two materials, one difference can be found. If the presence of functional groups is not implied in the SBS structure, then Nylon, on the contrary, initially has an amide group, which promotes the formation of additional hydrogen bonds to enhance adhesion.

## Conclusions

Manufacturing of acetylene using the carbide approach provides a significant amount of the CCR byproduct. The main component of CCR is calcium hydroxide, which negatively affects the environment due to its alkaline nature and reactivity. Many outstanding research endeavors have been dedicated to utilizing CCR as a filler in construction and building materials due to the better strength of the obtained materials.

In line with the requirements in this field, CCR was used in polymer dispersion-filled composite materials with improved strength characteristics in this work for the first time. New polymer composite materials were obtained by extruding commercially available plastics followed by 3D printing with CCR. CCR demonstrated compatibility with all the types of plastics studied, and the resulting composite wires can easily be used in 3D printing. Composite materials show improved properties compared to plastics. The best result was achieved using Nylon, where a sufficiently large amount of the filler (20 wt%) provided an increase in the material tensile strength and Young’s modulus by 9% and 60%, respectively. In the case of materials based on Nylon, SBS, ABS and HIPS, the printed parts at the end of life can be re-extruded and reused at least 3 times. The use of 20 wt% CCR in Nylon also reduced the shrinkage of the material by 5%.

SEM studies have shown the diverse nature of the interaction of the polymer matrix with the CCR filler. In the cases of ABS, HIPS, and PETG, a clear phase boundary was observed, and in the cases of Nylon and SBS, these boundaries are amorphous, which suggests a closer interaction of these plastics with the filler. A partial conversion of the CCR during preparation and extrusion was detected using XRD and EDX due to the capture of carbon dioxide from the atmosphere followed by the formation of CaCO_3_.

We anticipate that the developed approach can be helpful to build a sustainable and environment-protecting cycle for the calcium carbide lifecycle. Broad use of CCR-filled composites may be expected due to the rather small cost of this filler and compatibility with standard polymers. In addition to CCR utilization, the advantageous material features introduced in the composite structure are valuable for practical use.

## Supplementary Information

Supplementary Information.

## Data Availability

The datasets generated during and/or analyzed during the current study are included in this published article and its Supplementary Information files.
