# Supplementary material for: Sustainable application of calcium carbide residue as a filler for 3D printing materials
Source: Sci Rep. 2023 Mar 17;13:4465. doi: 10.1038/s41598-023-31075-z (PMC10023683; doi:10.1038/s41598-023-31075-z)
Supplement: Supplementary file 1 — Supplementary Information. [file 41598_2023_31075_MOESM1_ESM.pdf]

Supporting information

Sustainable application of calcium carbide residue as a filler for 3D printing materials

Dmitriy E. Samoylenko[1], Konstantin S. Rodygin[1] and Valentine P. Ananikov*[1,2]

[1]Saint Petersburg State University, Universitetskiy pr. 26, Stary Petergof 198504, Russia.

[2]N. D. Zelinsky Institute of Organic Chemistry, Russian Academy of Sciences, Leninsky pr. 47, Moscow 119991, Russia. *E-mail: val@ioc.ac.ru.

Contents

S1. 3D MODELS OF THE PRINTED OBJECTS S3

S2. 3D PRINTING CONDITIONS OPTIMIZATION S4

S3. SEM INVESTIGATIONS S5

S4. 3D PRINTED SAMPLES S21

S5. XRD INVESTIGATION OF CCR OBTAINED S23

S6. QUALITY TESTS OF THE PRINTED SAMPLES S24

S7. TENSILE TESTS S26

S1. 3D models of the printed objects

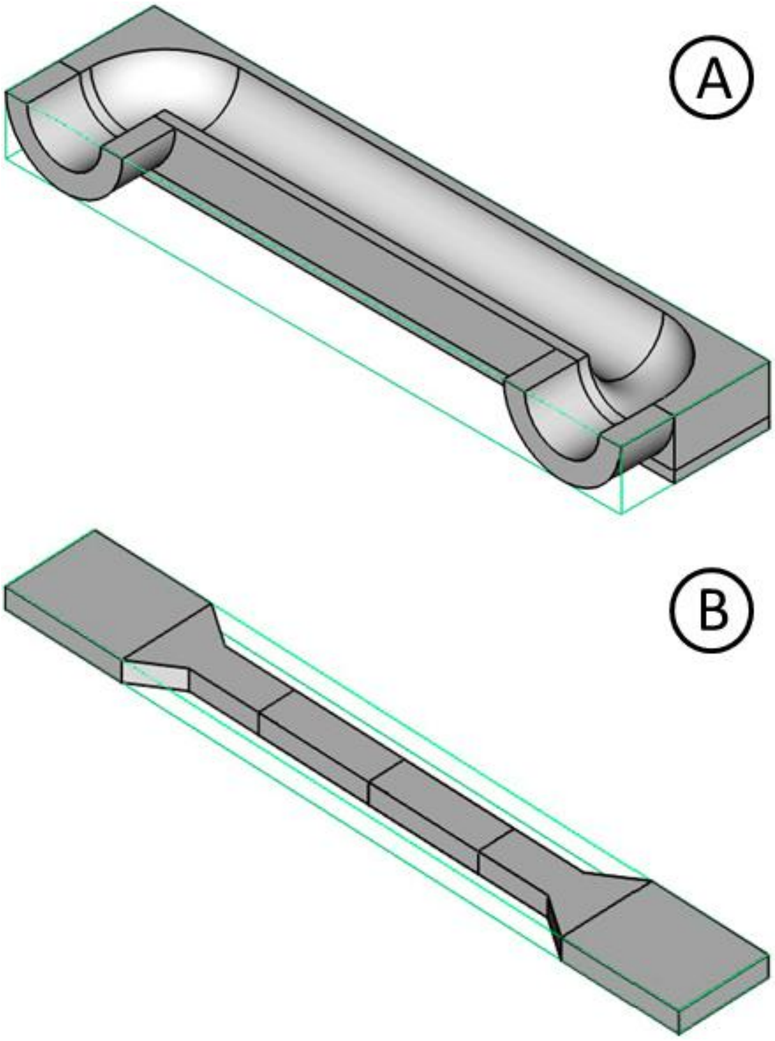

Figure S1. Printed 3D models for shrinkage tests (A) and tensile strength analysis (B).

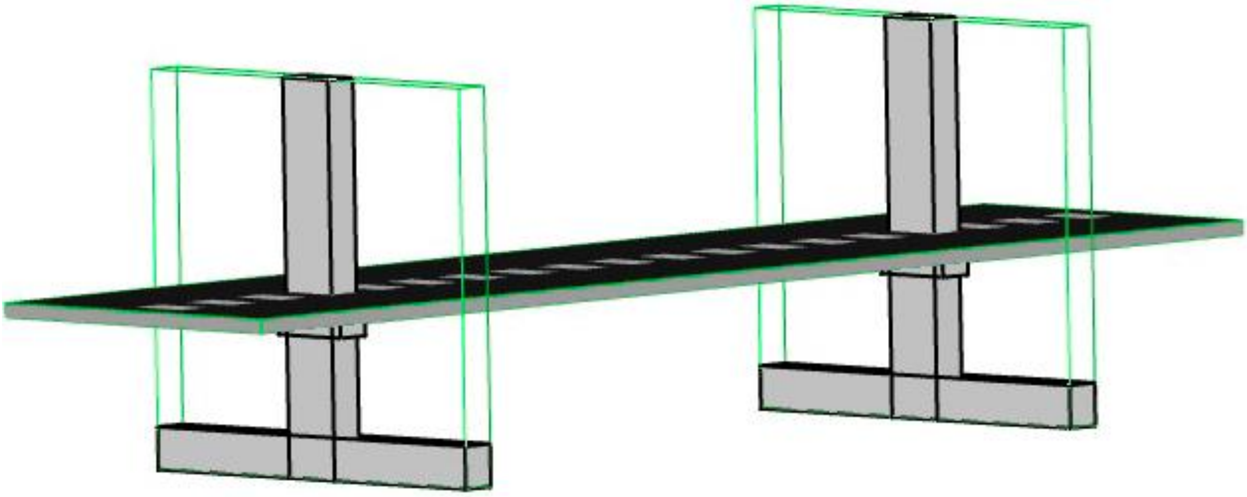

Figure S2. Printed 3D models for shrinkage evaluation.

S2. 3D printing conditions optimization

It should be noted that optimal printing conditions depend on the 3D printer model. At the first stage, the printing conditions for pure plastic, declared by the manufacturer, were checked (Table S1.). In the case of Nylon, the manufacturer recommends using a nozzle temperature of 230-270 °C during the printing process, while the build platform temperature should be 70-90 °C. Based on this, the initial temperature of the nozzle is 230 °C, and the temperature of the build platform is 70 °C. The printing speed, cooling intensity and extrusion multiplier corresponded to the standard slicer (Maestro Wizard 3.6.0) settings and were 30 mm/sec, 105% and 0%, respectively (entry 1). Five samples were printed at the same time to meet the same conditions. Thus, in the process of printing, some of the samples were partially detached from the surface of the build platform due to shrinkage. This leads to displacement of subsequent layers of the part or to complete tearing off of the part with inevitable damage to the integrity of the remaining samples. To improve the adhesion of the first printed layer to the build platform, we increased the temperature of the table in steps by 10 °C (entries 2-4). By increasing the temperature up to 100 °C, we managed to achieve strong adhesion of the printed part to the build platform. However, the printed parts had distorted geometry, and excess plastic was observed on the edges of the samples. To solve this problem, we reduced the extrusion multiplier by 5%, which significantly improved the quality of the finished part (entry 5). These conditions were considered optimal for printing parts using Nylon. It should be noted that Nylon is very sensitive to air moisture and requires pre-drying before printing. Without this stage, there is a high probability of bubble formation due to the evaporation of adsorbed moisture, which significantly affects the strength characteristics and overall dimensions of the target product.

The nylon-based composites were also predried to produce the highest quality prints. The starting point for optimizing print conditions was the optimum print conditions for transparent plastic (entry 6). The finished samples had a significant broadening in the lower layers; in addition, the outer edges had protruding areas, indicating an excess of material supplied to the print head. The expansion of the lower part in the sample occurs due to the pressure of the upper layers, while the lower layers have not cooled enough to retain their shape. To exclude this defect, the temperatures of the build platform and nozzle (entry 7) were reduced, which allowed the lower layers to cool faster and not deform during printing. In this case, the adhesion of the sample to the surface of the build platform was sufficient to obtain a high-quality part. The excess media issue was addressed by reducing the extrusion multiplier and print speed (entry 8). Similarly, optimal printing conditions were found for other materials used.

Table S1. Optimizing printing conditions using Nylon based materials as an example.

Entry	Material	Temperature, °C		Cooling Intensity, %	Extrusion Multiplier, %	Printing speed, mm/sec
		Build Platform	Noozle			
1	Nylon	70	230	0	105	30
2		80	230	0	105	30
3		90	230	0	105	30
4		100	230	0	105	30
**5**		**100**	**230**	**0**	**100**	**30**
6	Nylon-CCR	100	230	0	100	30
7		65	225	0	100	30
**8**		**65**	**225**	**0**	**98**	**20**

S3. SEM investigations

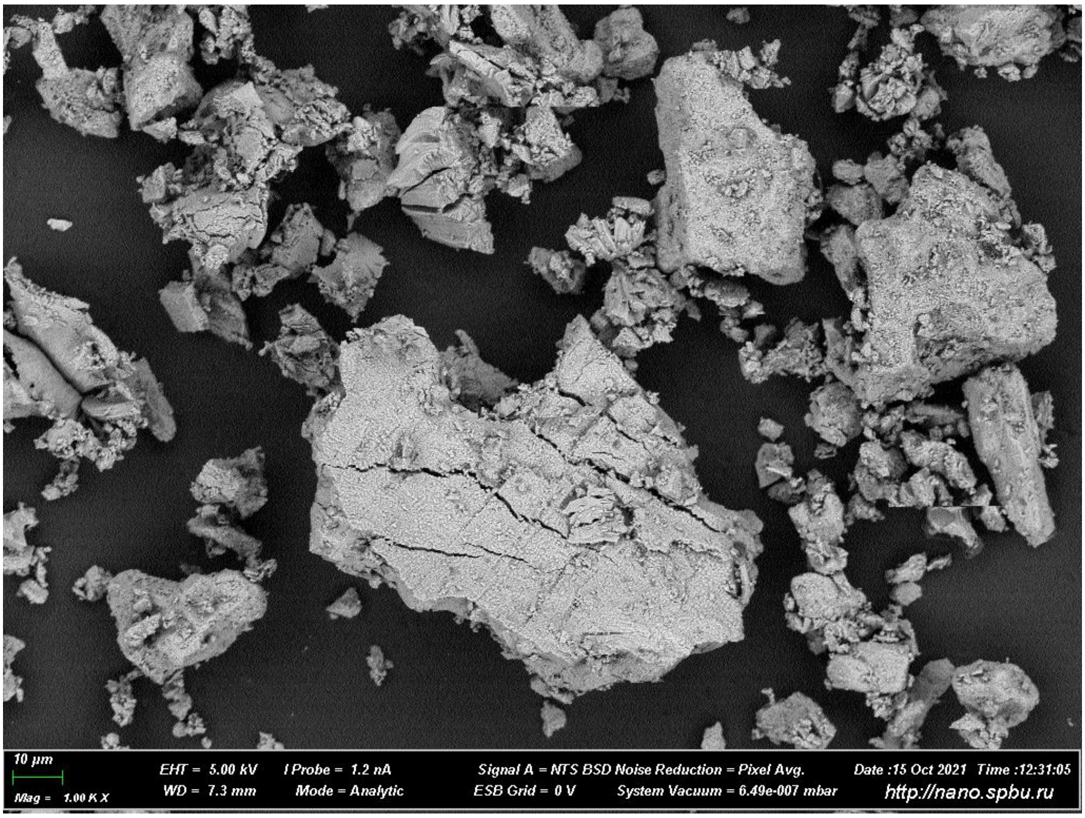

Figure S3. SEM image of CCR particles. Scale bar is 10 µm.

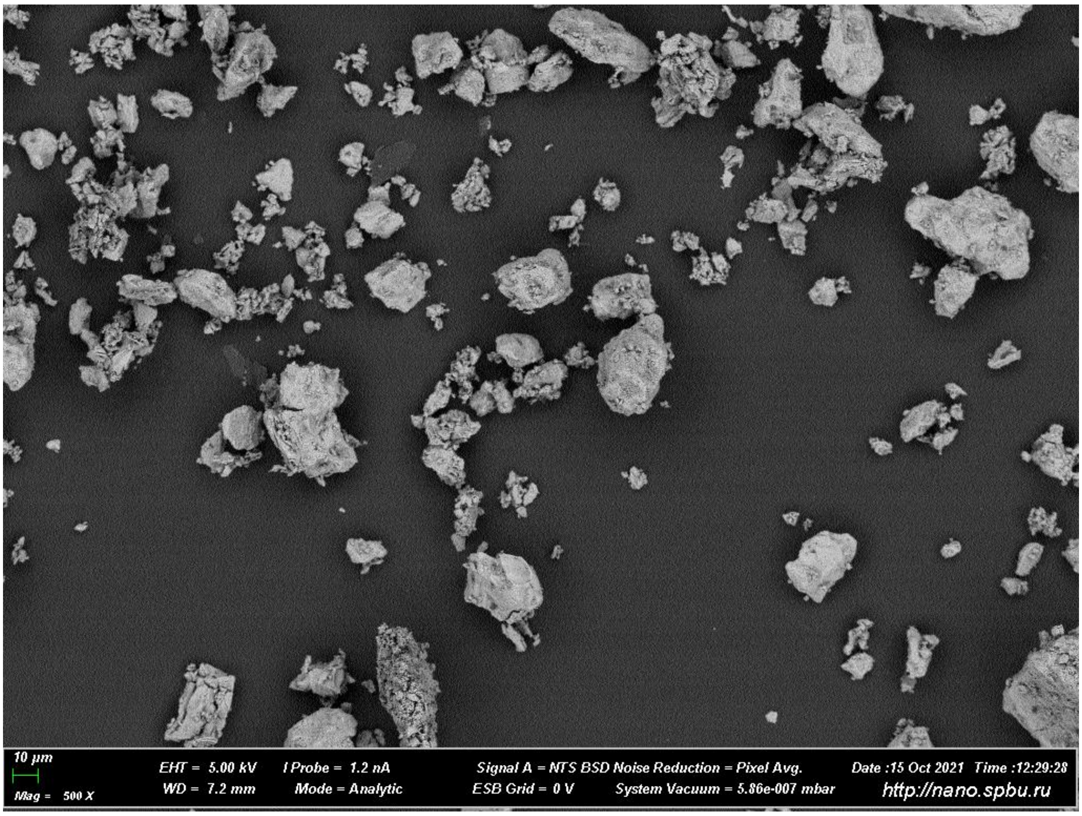

Figure S4. SEM image of CCR particles for aggregation studies. Scale bar is 10 µm.

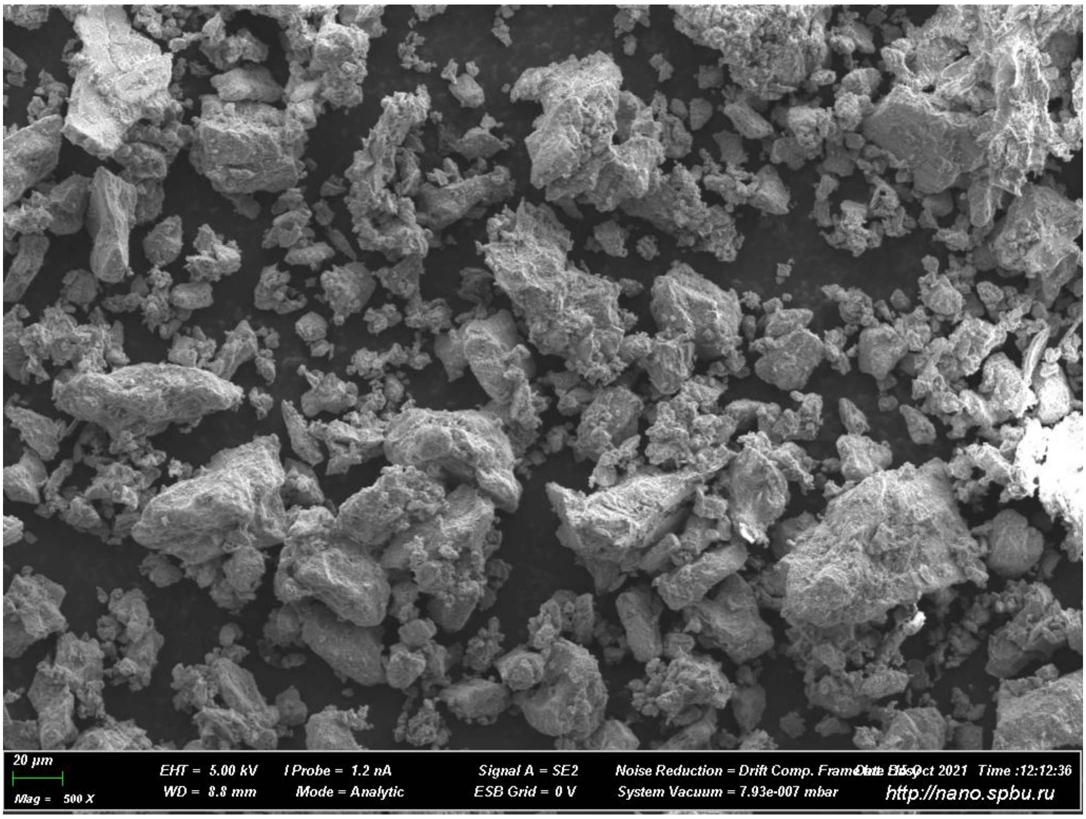

Figure S5. SEM image of CCR particles. Scale bar is 20 µm.

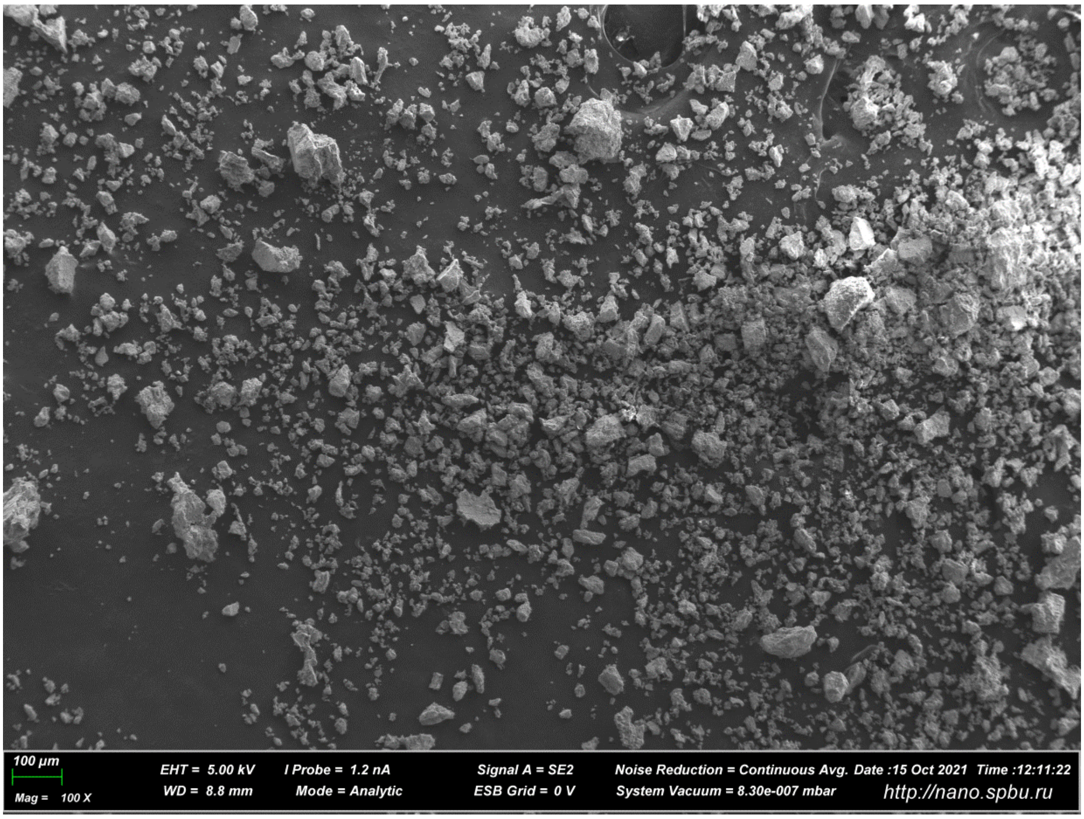

Figure S6. SEM image of CCR particles. Scale bar is 100 µm.

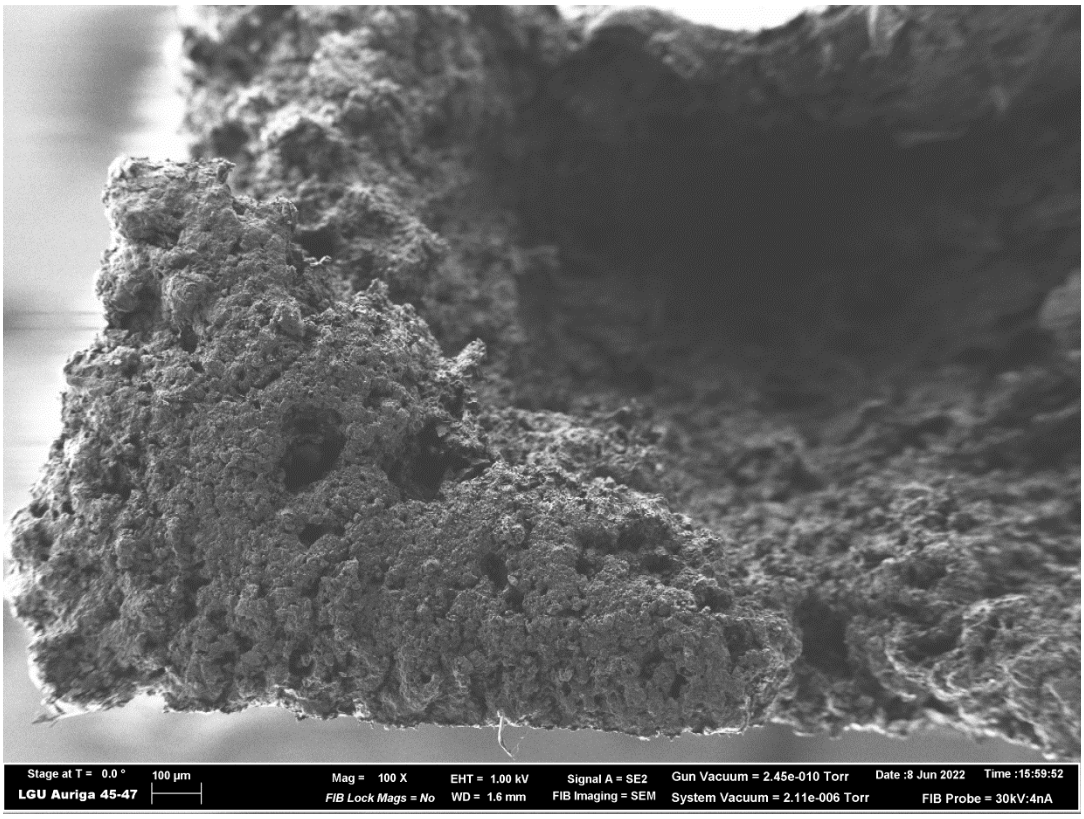

Figure S7. SEM image of the 3D printed paddle (Nylon and 20 mas.% of CCR) after tensile tests. Scale bar is 100 µm.

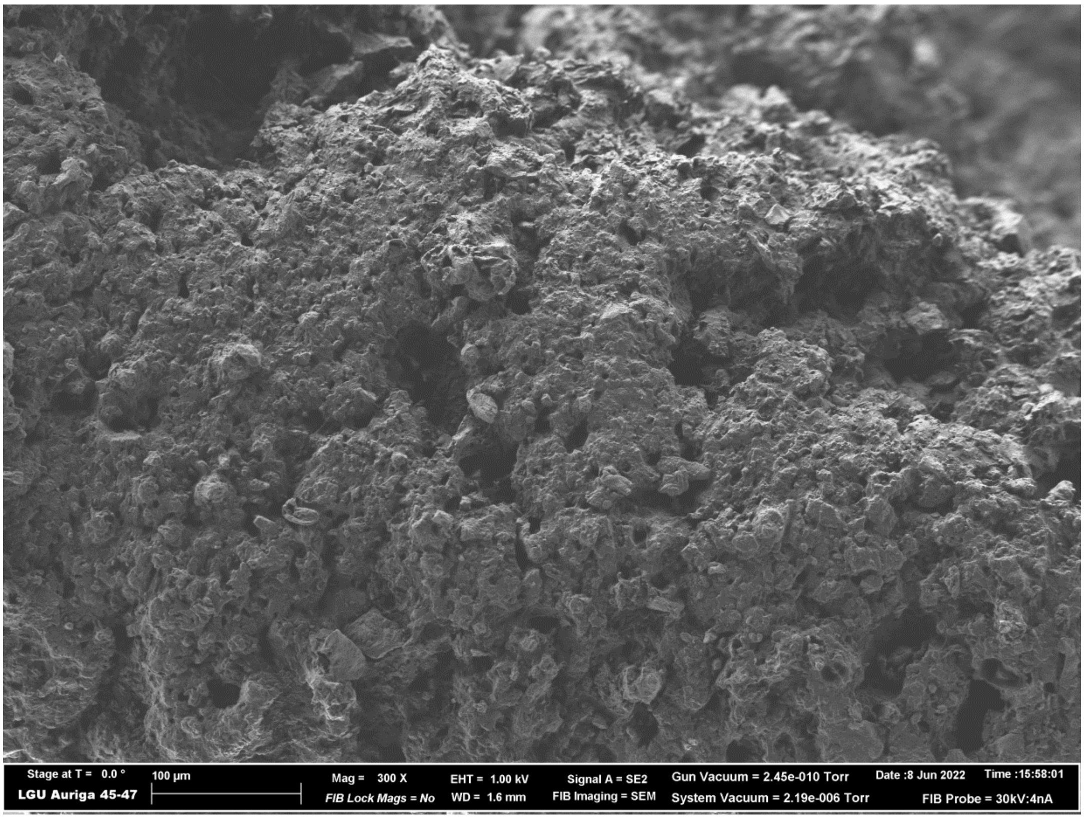

Figure S8. SEM image of the 3D printed paddle (Nylon and 20 mas.% of CCR) after tensile tests. Scale bar is 100 µm.

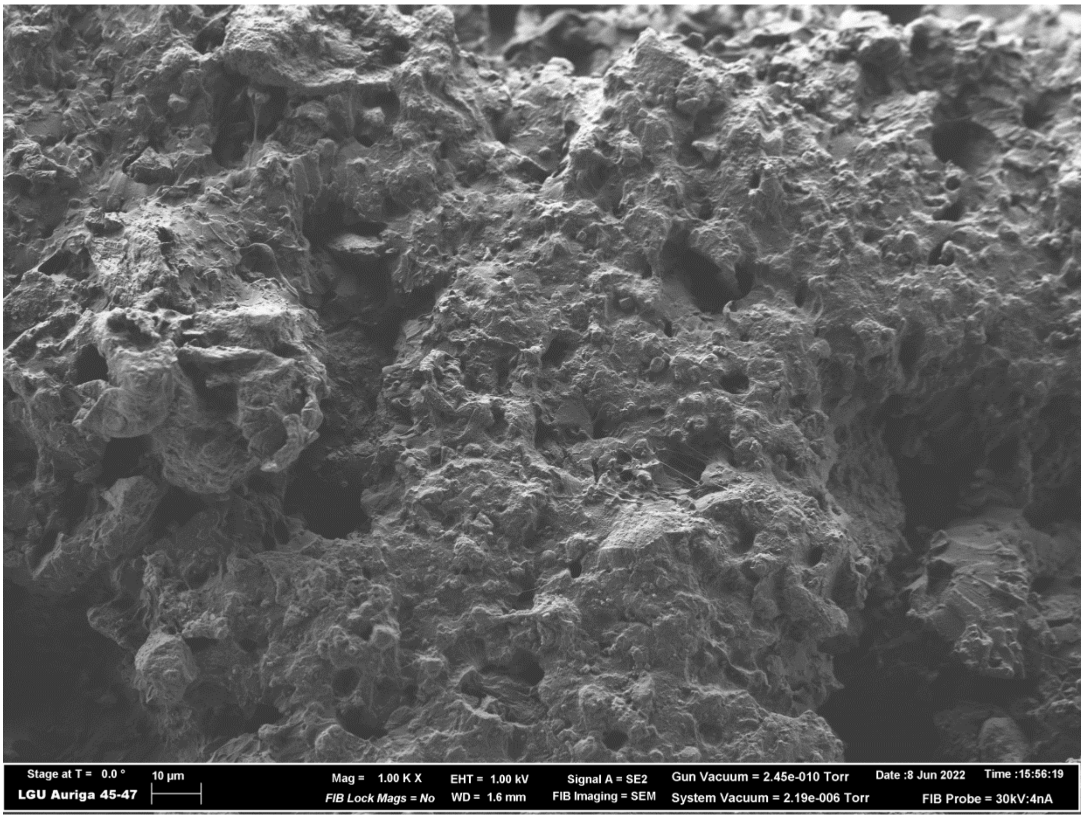

Figure S9. SEM image of the 3D printed paddle (Nylon and 20 mas.% of CCR) after tensile tests. Scale bar is 10 µm.

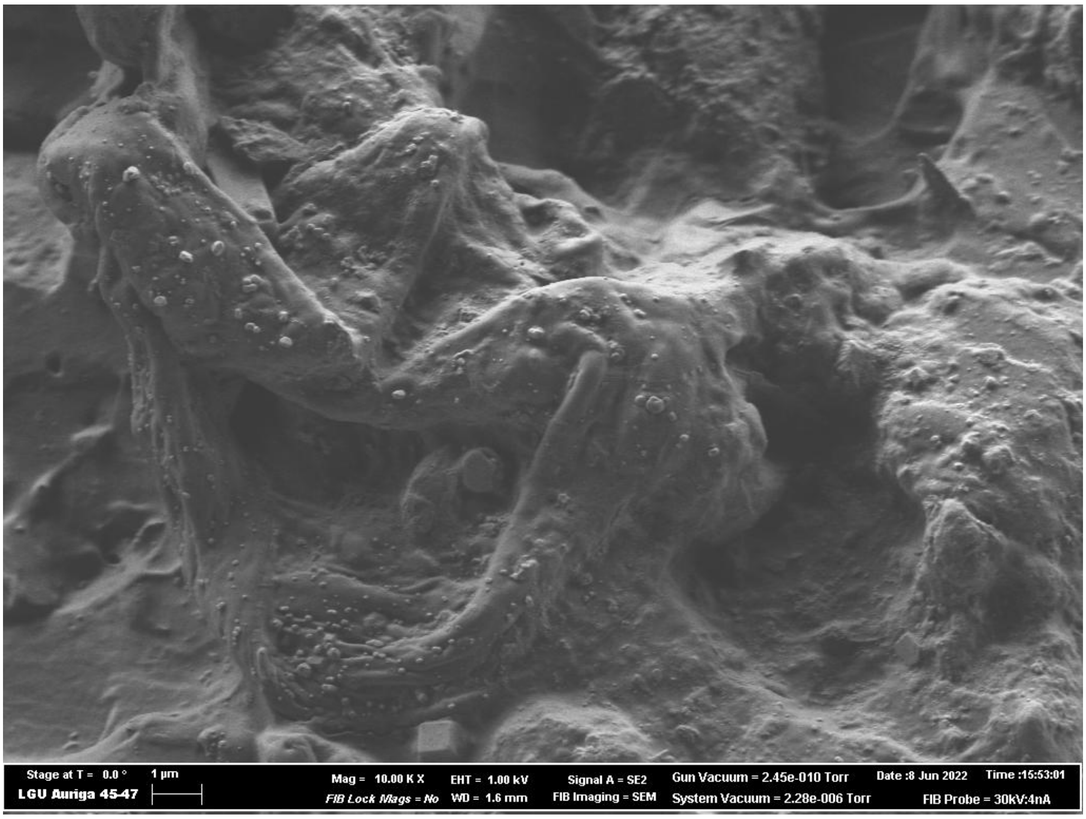

Figure S10. SEM image of the 3D printed paddle (Nylon and 20 mas.% of CCR) after tensile tests. Scale bar is 1 µm.

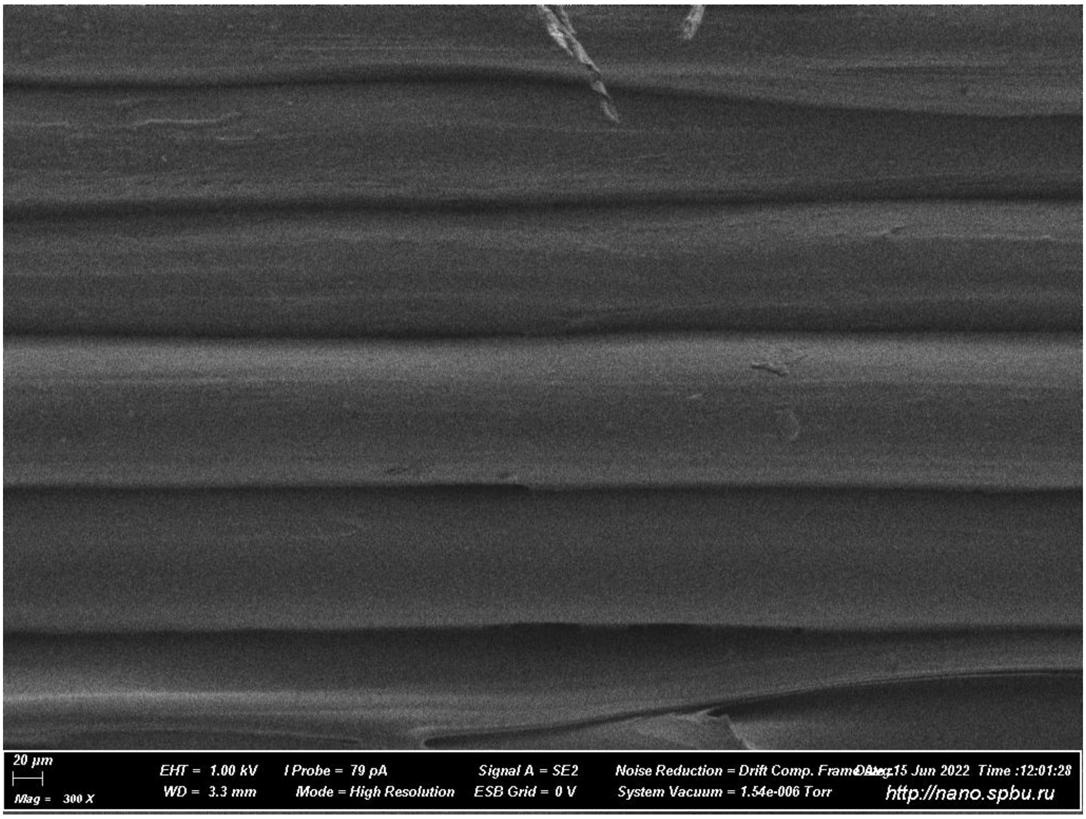

Figure S11. SEM image of the 3D printed paddle with neat Nylon. Scale bar is 20 µm.

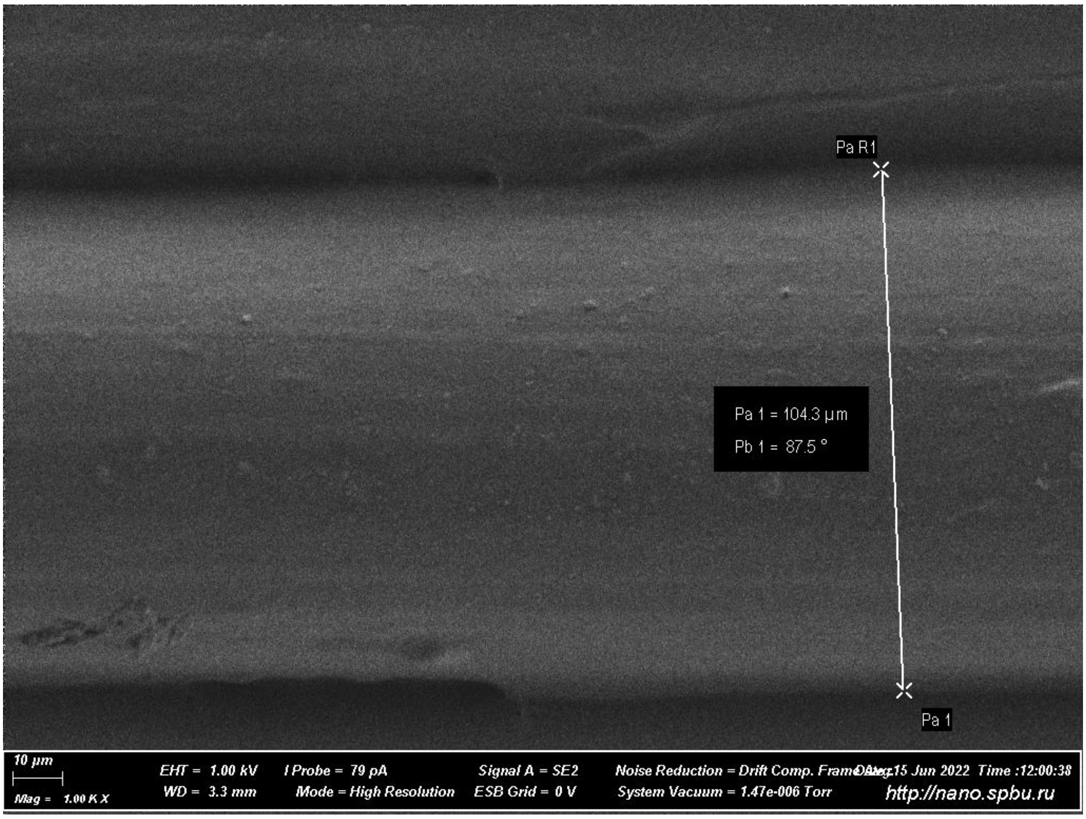

Figure S12. SEM image of the 3D printed paddle with neat Nylon. Scale bar is 10 µm.

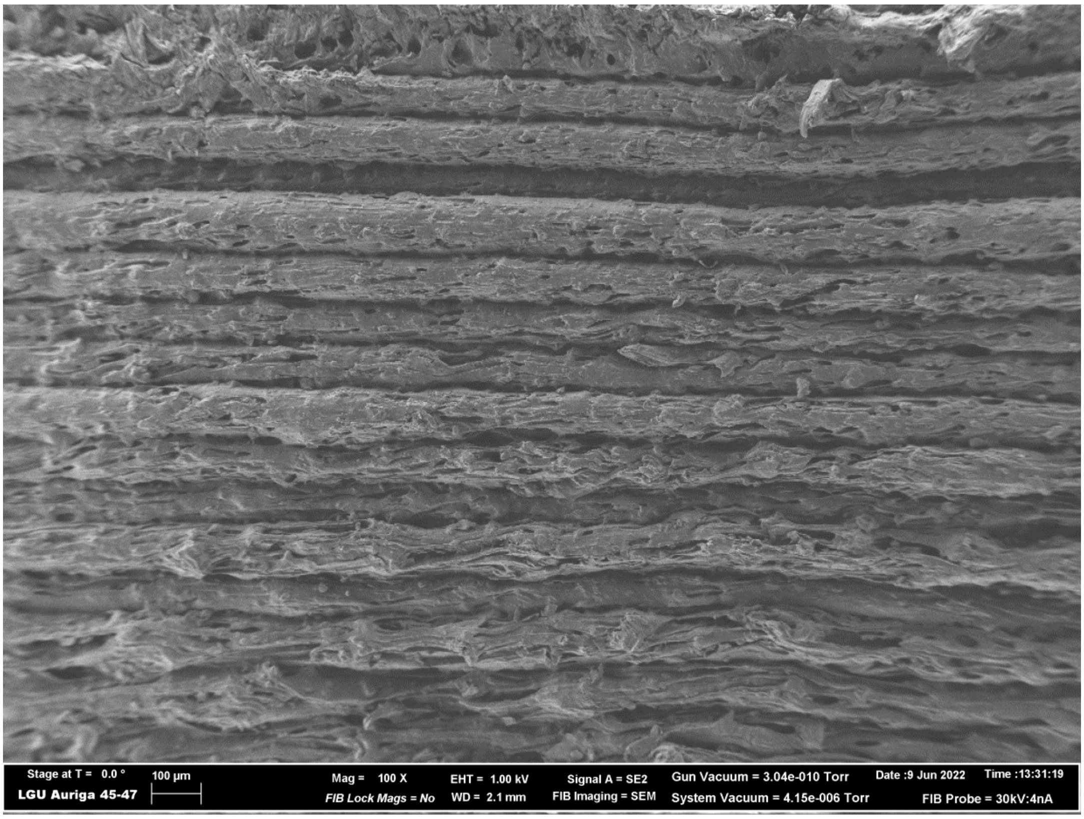

Figure S13. SEM image of the 3D printed paddle with Nylon composite (Nylon and 20 mas.% of CCR). Scale bar is 100 µm.

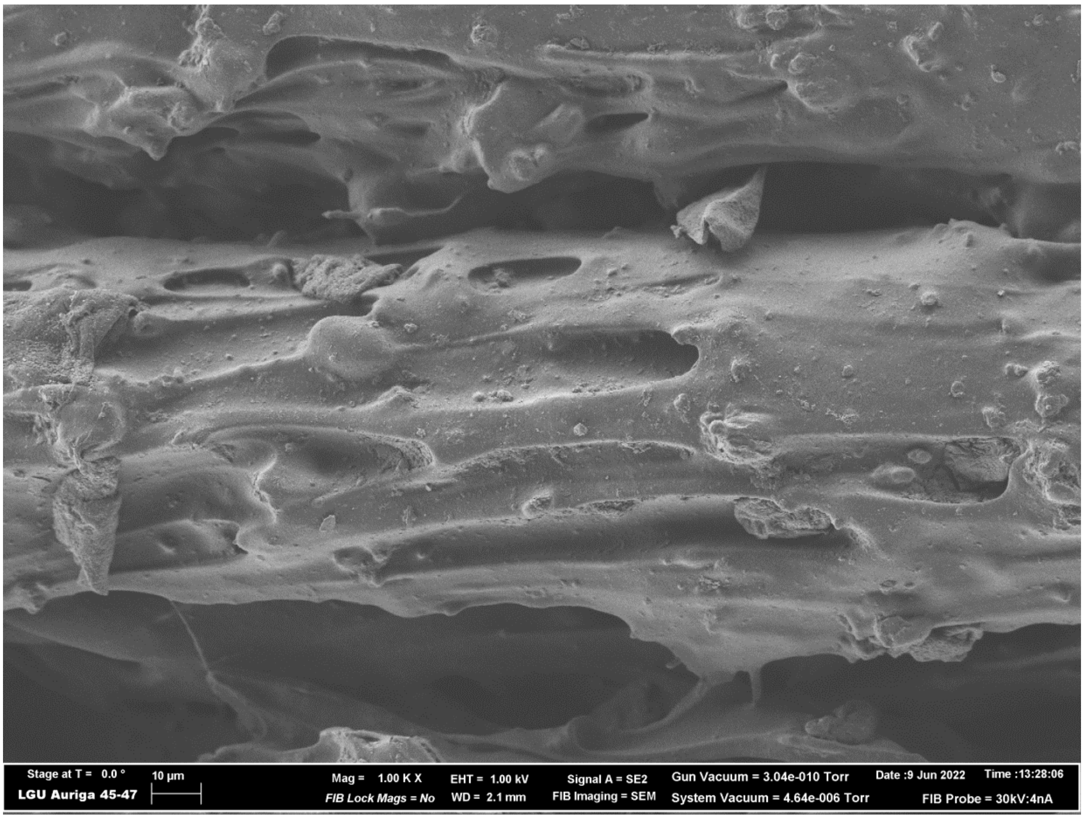

Figure S14. SEM image of the 3D printed paddle with Nylon composite (Nylon and 20 mas.% of CCR). Scale bar is 10 µm.

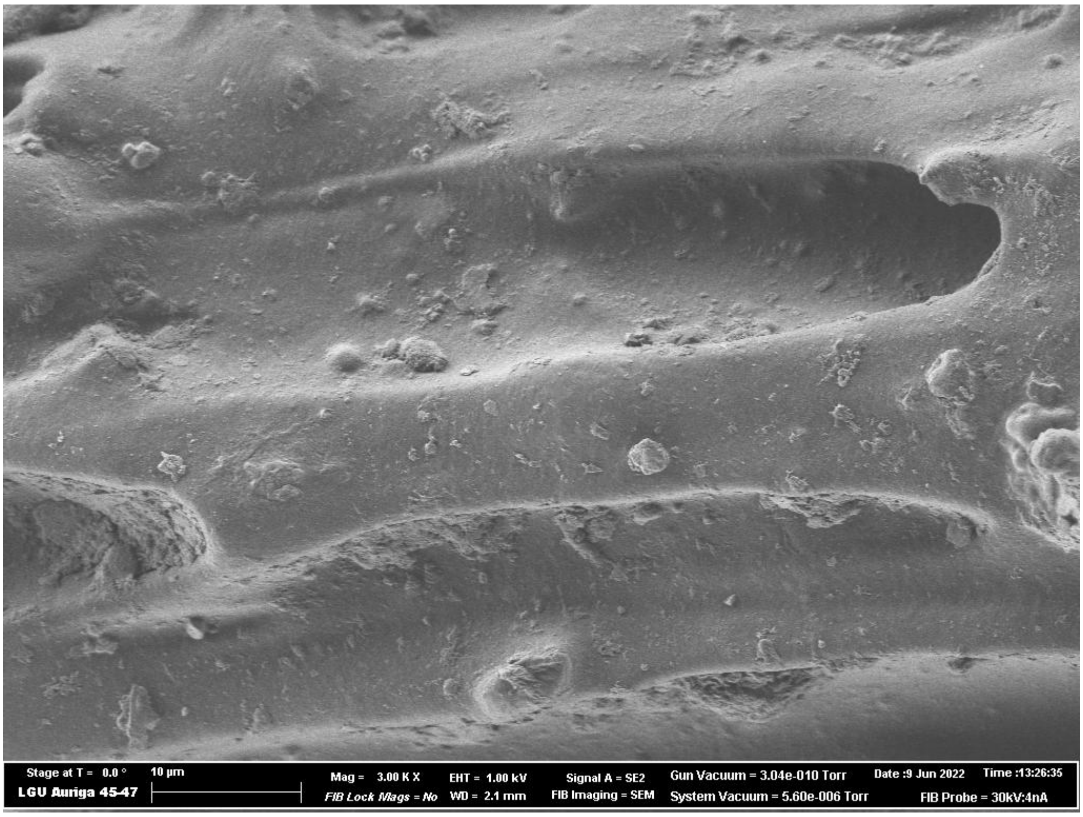

Figure S15. SEM image of the 3D printed paddle with Nylon composite (Nylon and 20 mas.% of CCR). Scale bar is 10 µm.

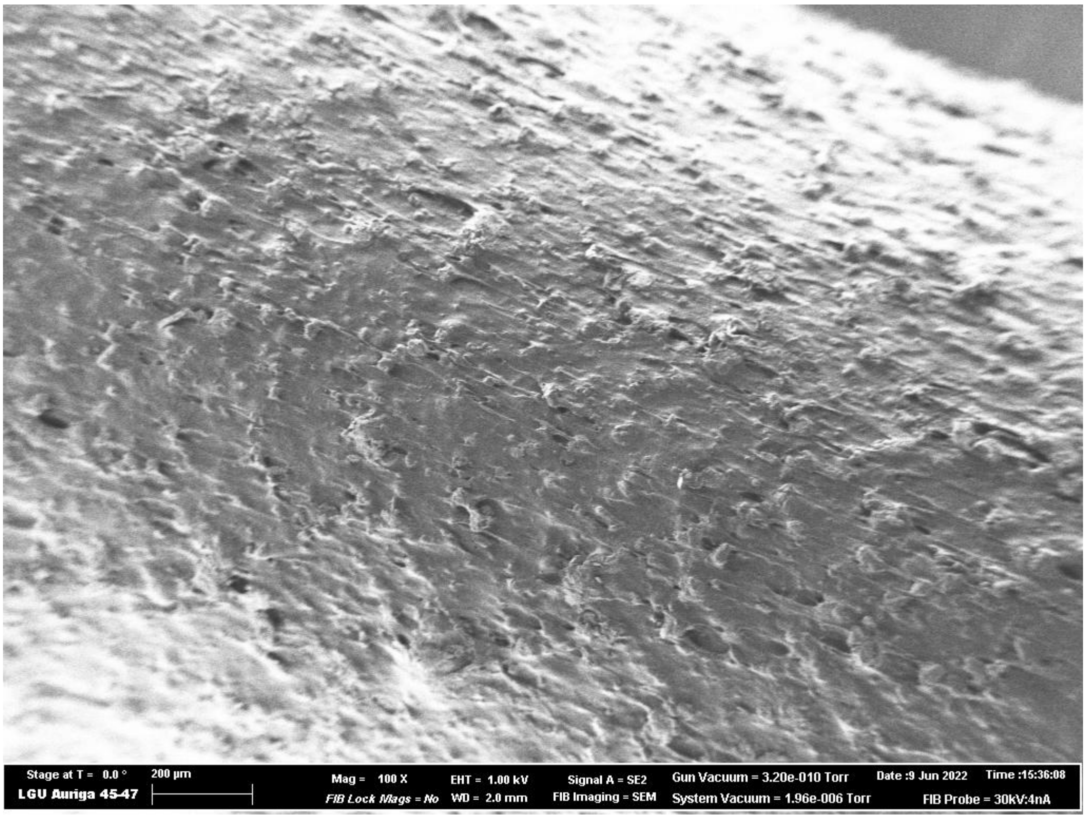

Figure S16. SEM image of the composite filament (Nylon and 20 mas.% of CCR) after extrusion. Scale bar is 200 µm.

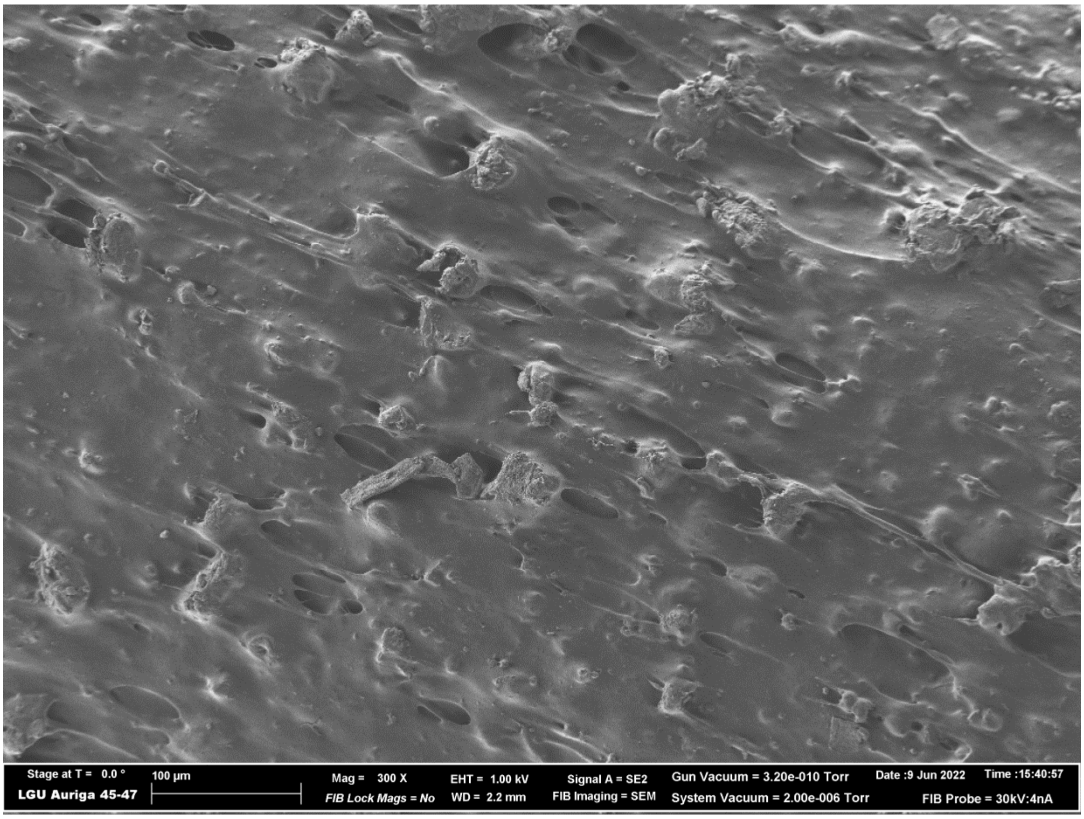

Figure S17. SEM image of the composite filament (Nylon and 20 mas.% of CCR) after extrusion. Scale bar is 100 µm.

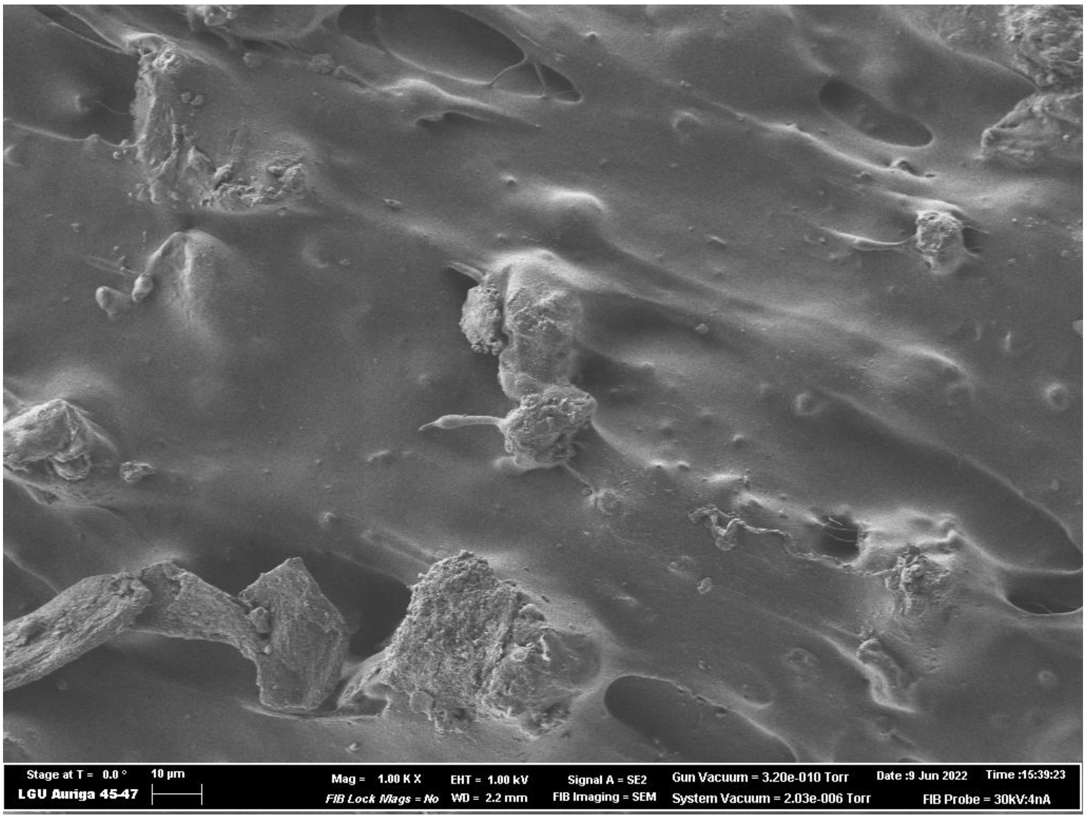

Figure S18. SEM image of the composite filament (Nylon and 20 mas.% of CCR) after extrusion. Scale bar is 10 µm.

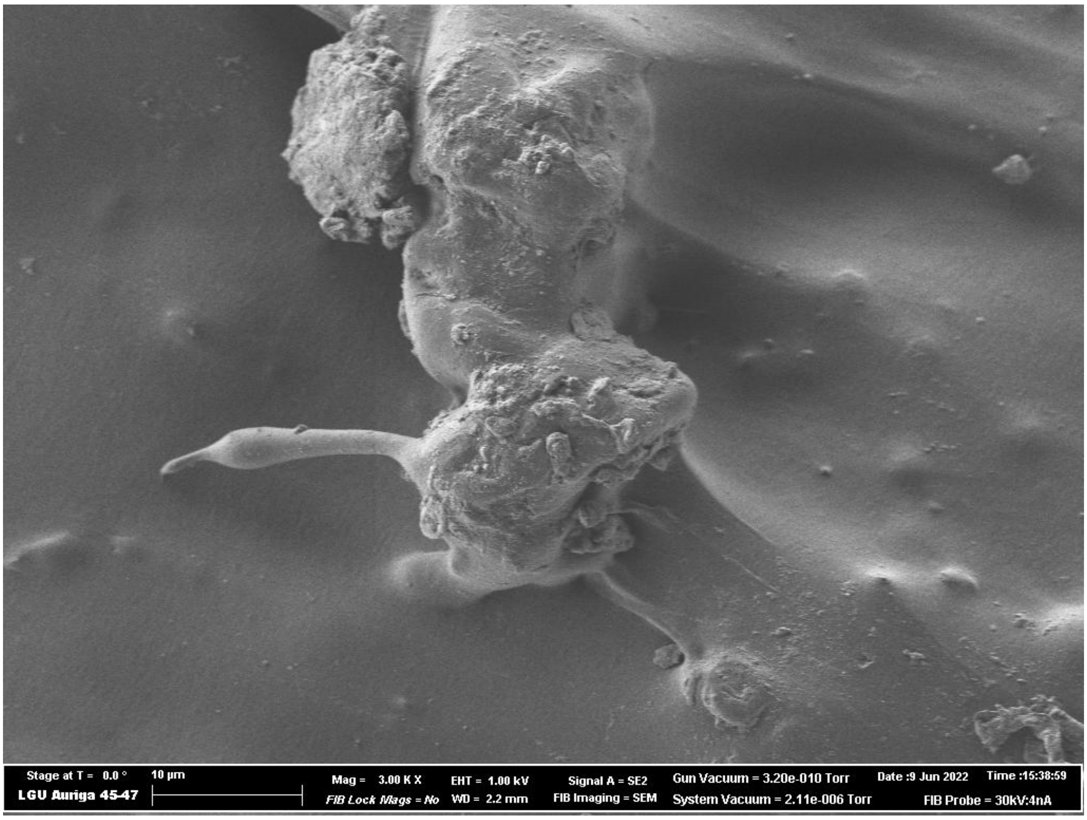

Figure S19. SEM image of the composite filament (Nylon and 20 mas.% of CCR) after extrusion. Scale bar is 10 µm.

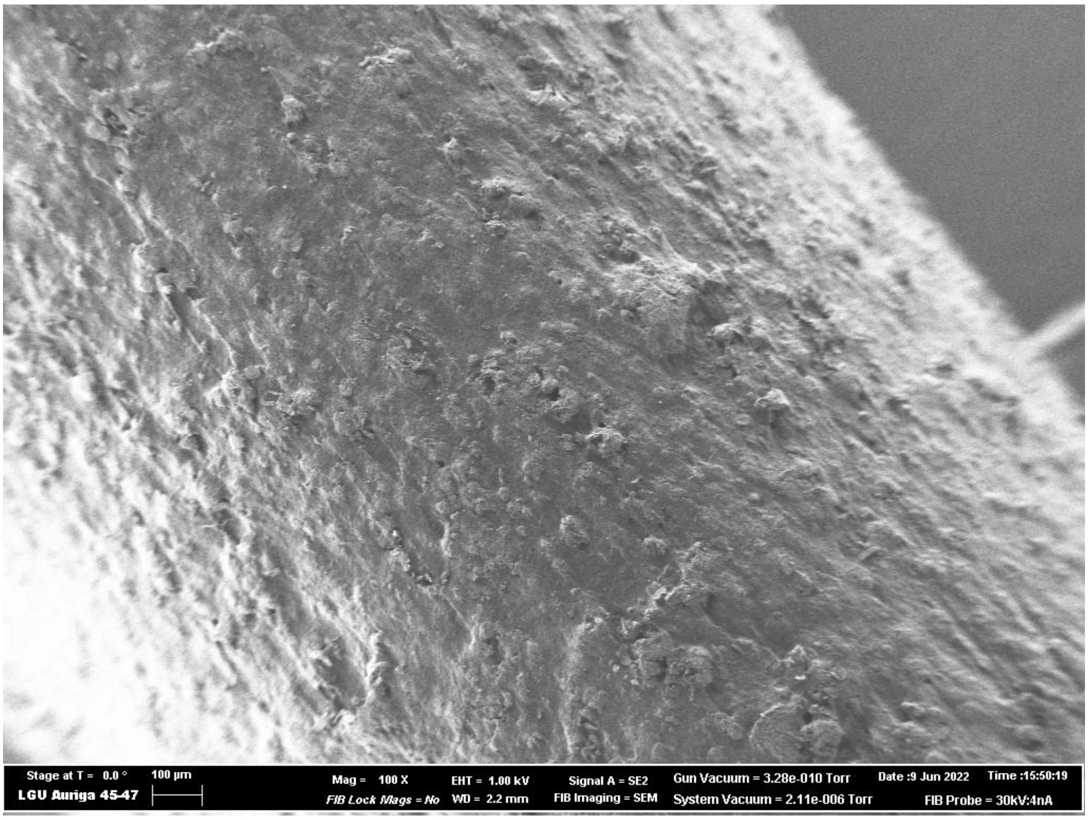

Figure S20. SEM image of the composite filament (Nylon and 28 mas.% of CCR) after extrusion. Scale bar is 100 µm.

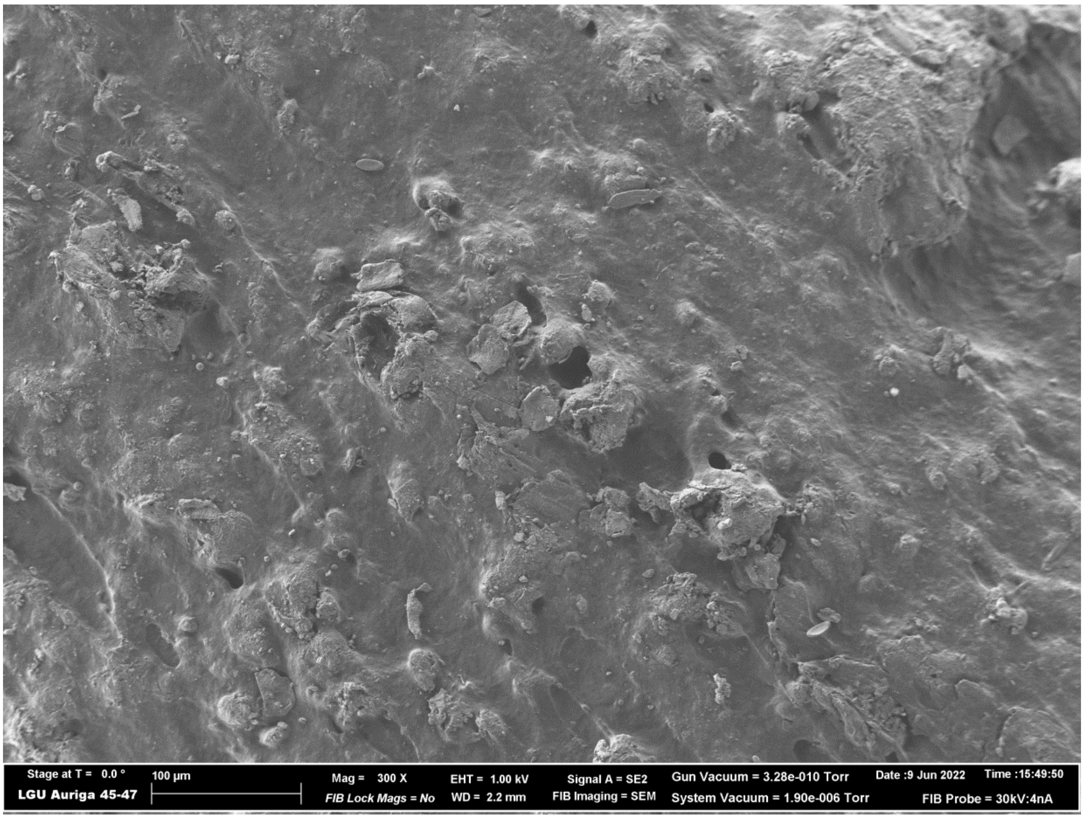

Figure S21. SEM image of the composite filament (Nylon and 28 mas.% of CCR) after extrusion. Scale bar is 100 µm.

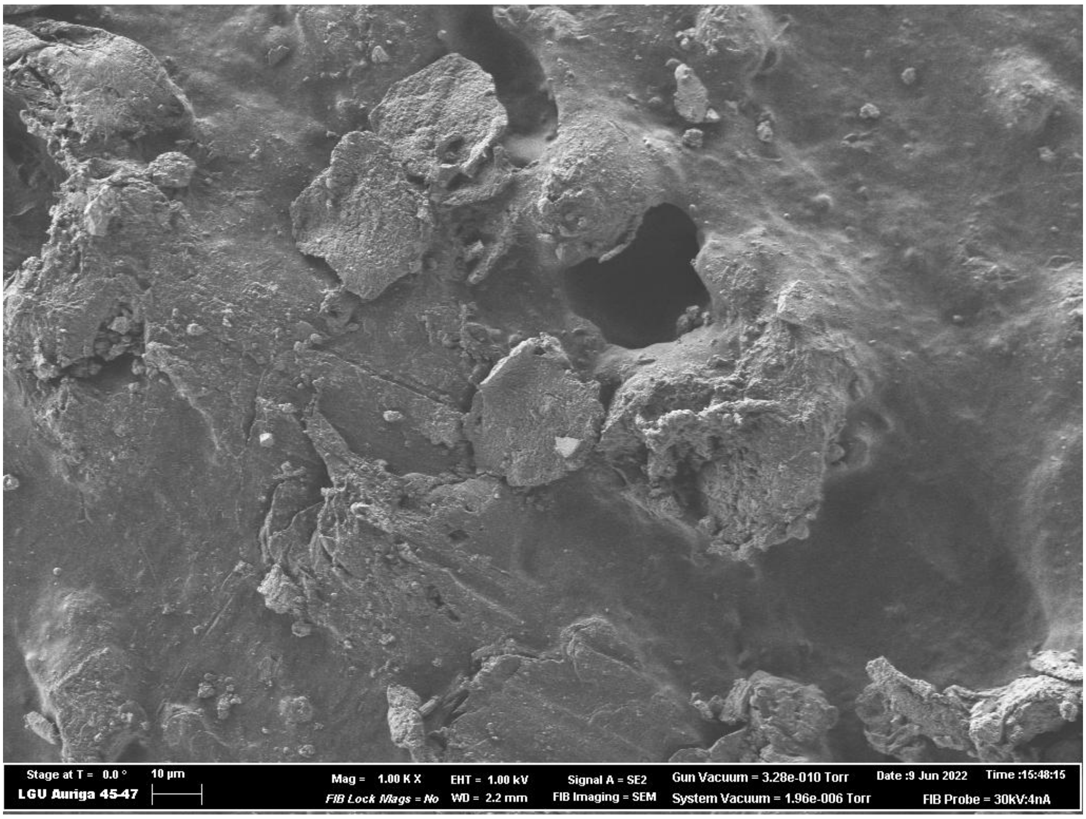

Figure S22. SEM image of the composite filament (Nylon and 28 mas.% of CCR) after extrusion. Scale bar is 10 µm.

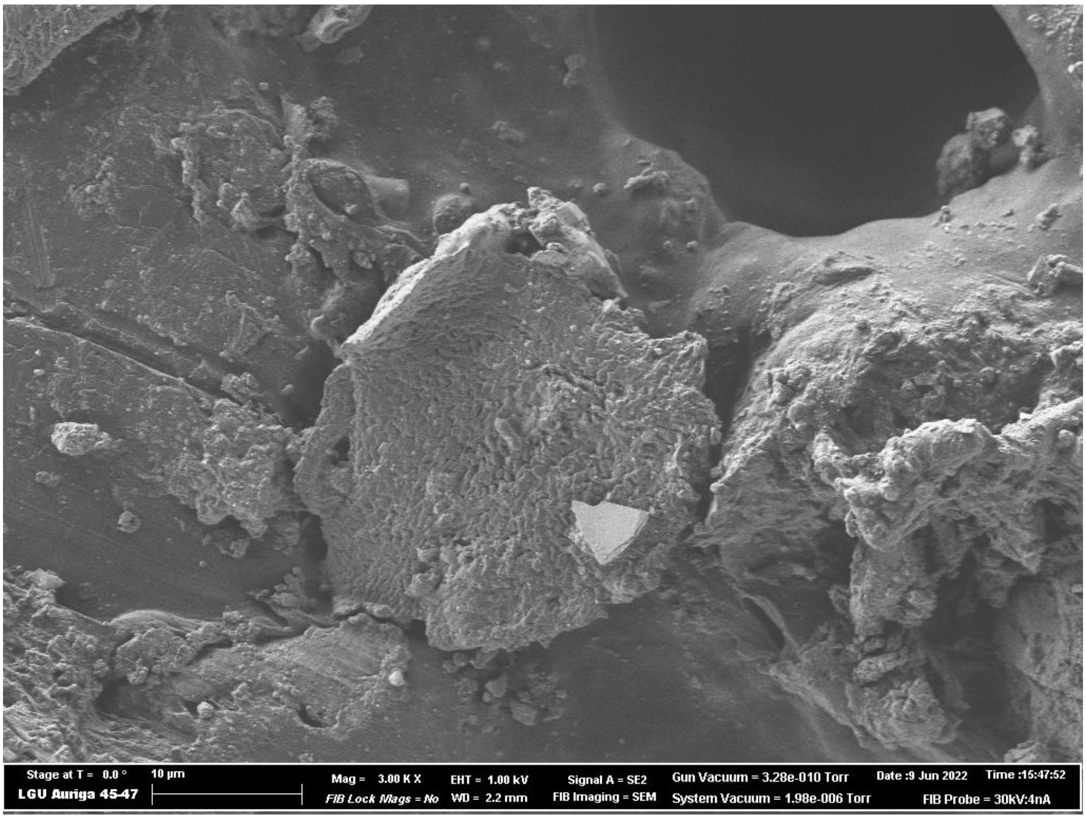

Figure S23. SEM image of the composite filament (Nylon and 28 mas.% of CCR) after extrusion. Scale bar is 10 µm.

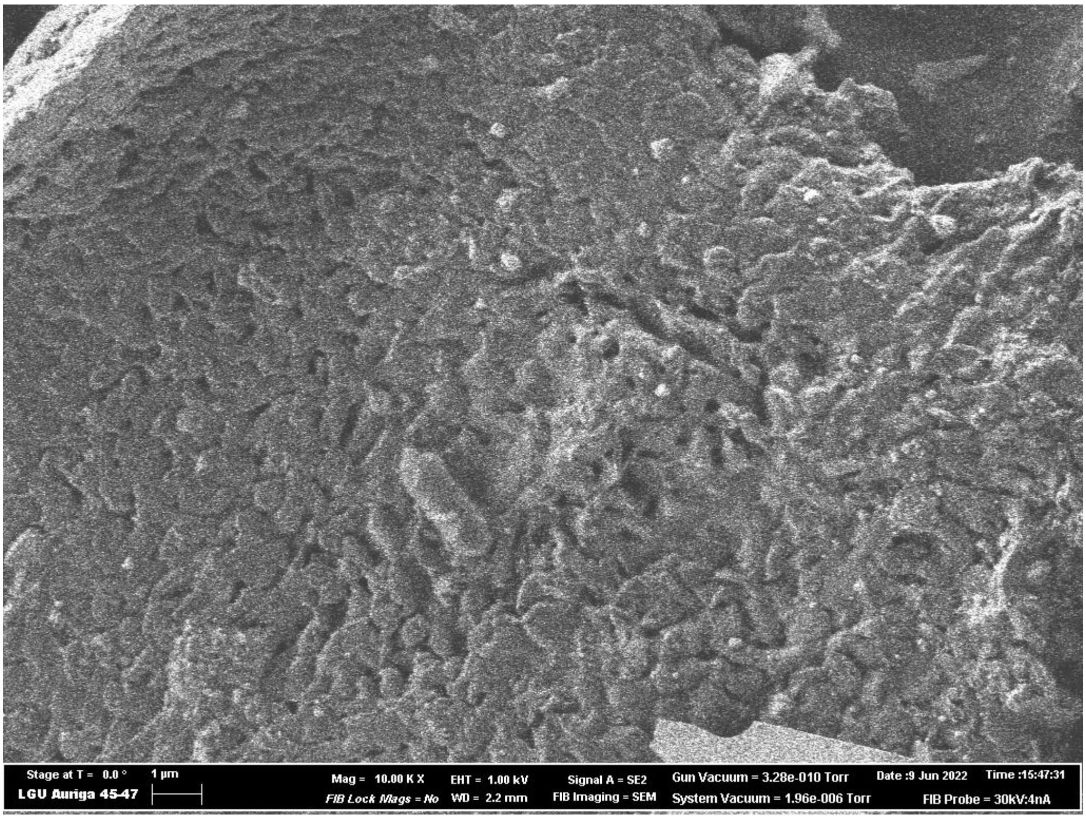

Figure S24. SEM image of the composite filament (Nylon and 28 mas.% of CCR) after extrusion. Scale bar is 1 µm.

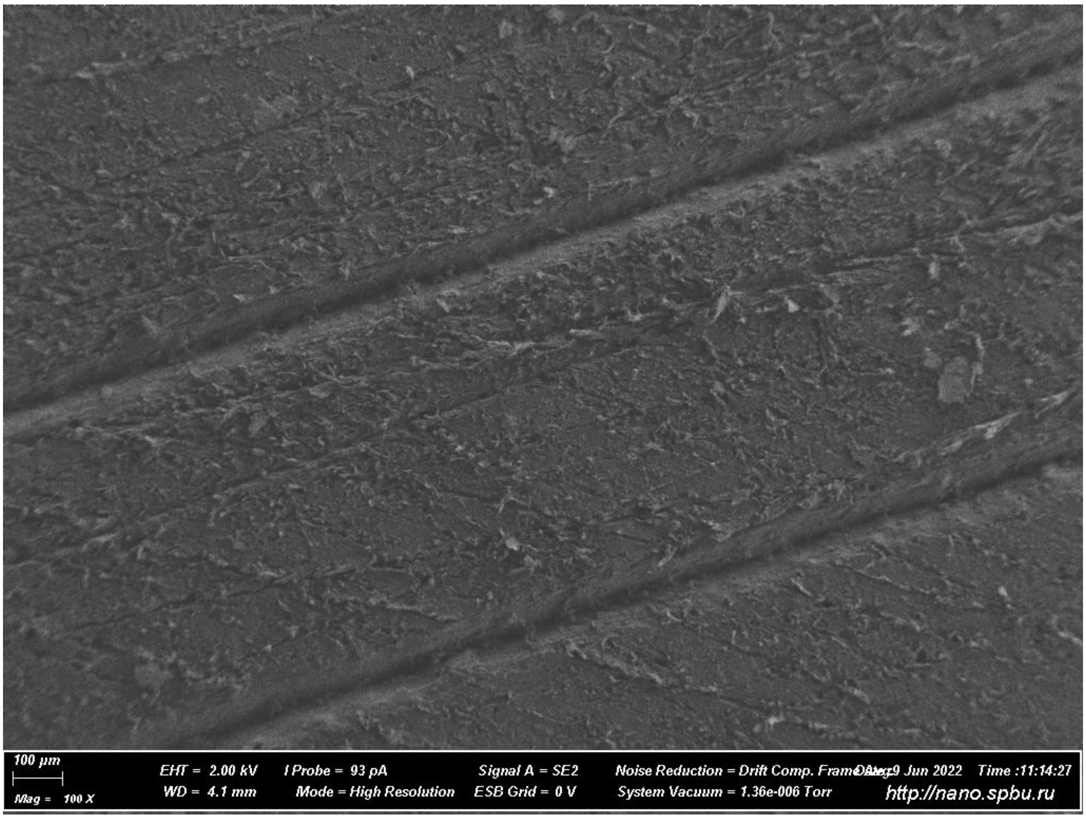

Figure S25. SEM image of the single layer printed with composite (Nylon and 20 mas.% of CCR). Scale bar is 100 µm.

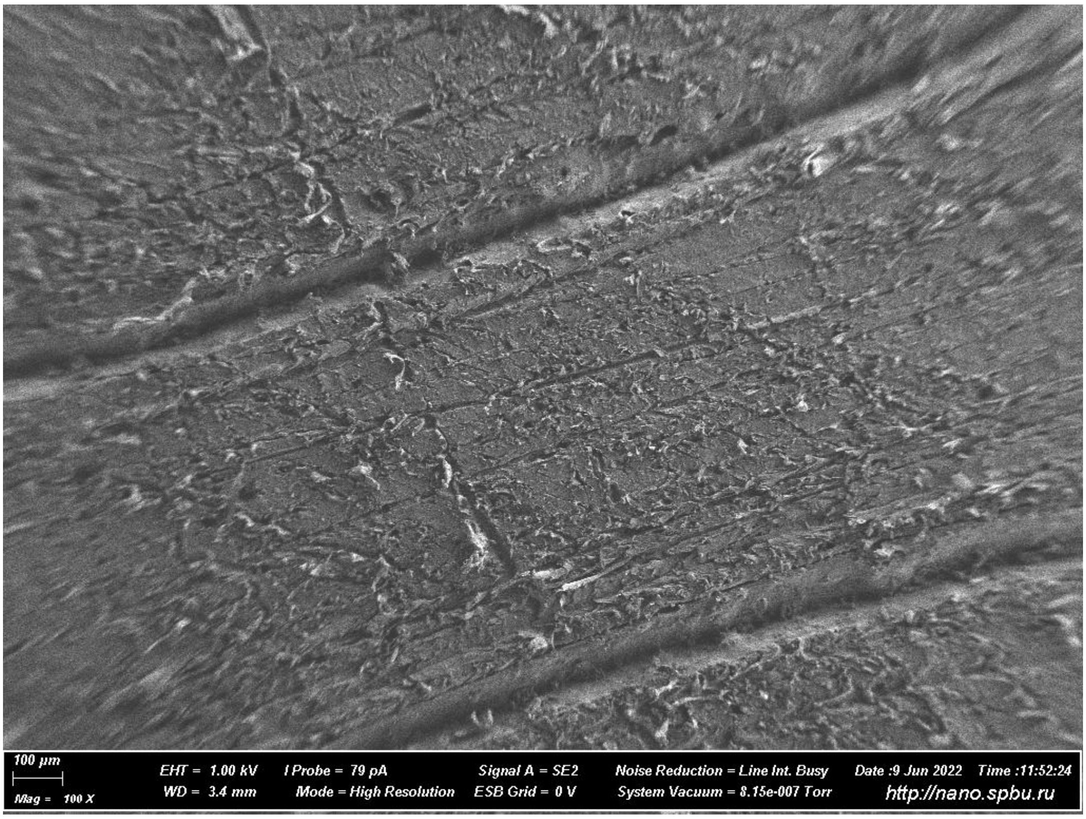

Figure S26. SEM image of the single layer printed with composite (Nylon and 20 mas.% of CCR). Scale bar is 100 µm.

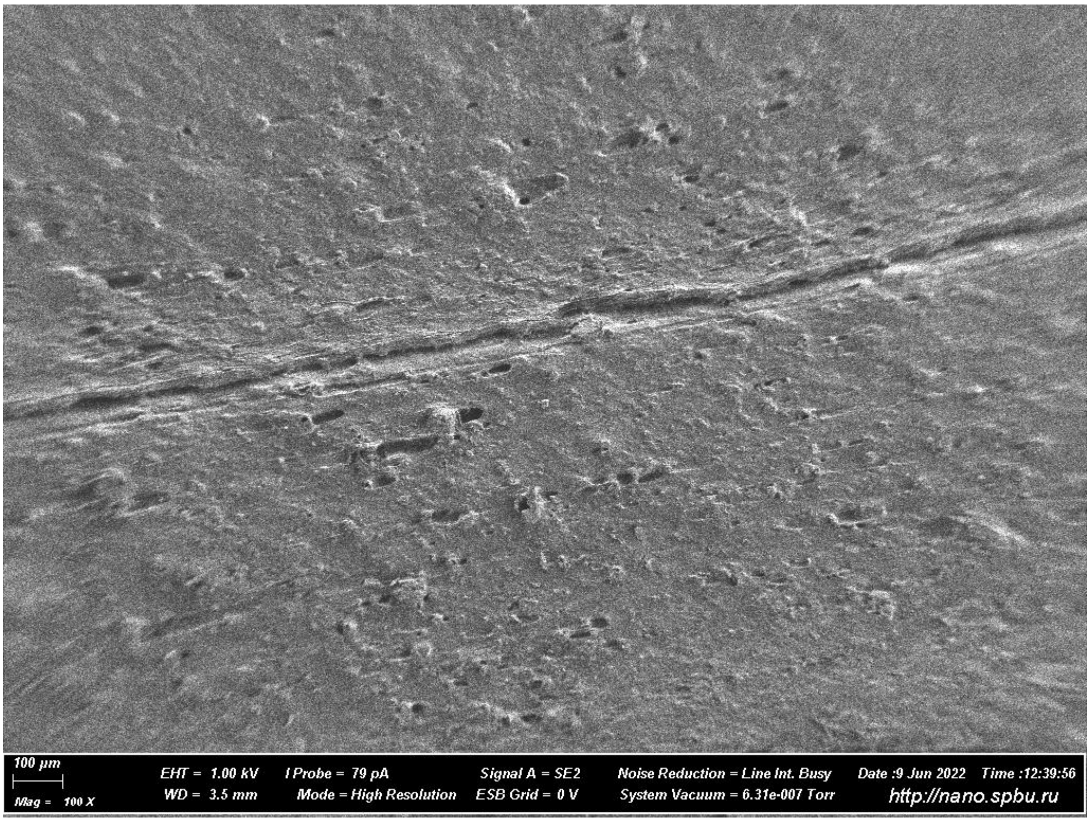

Figure S27. SEM image of the single layer printed with composite (Nylon and 20 mas.% of CCR). Scale bar is 100 µm.

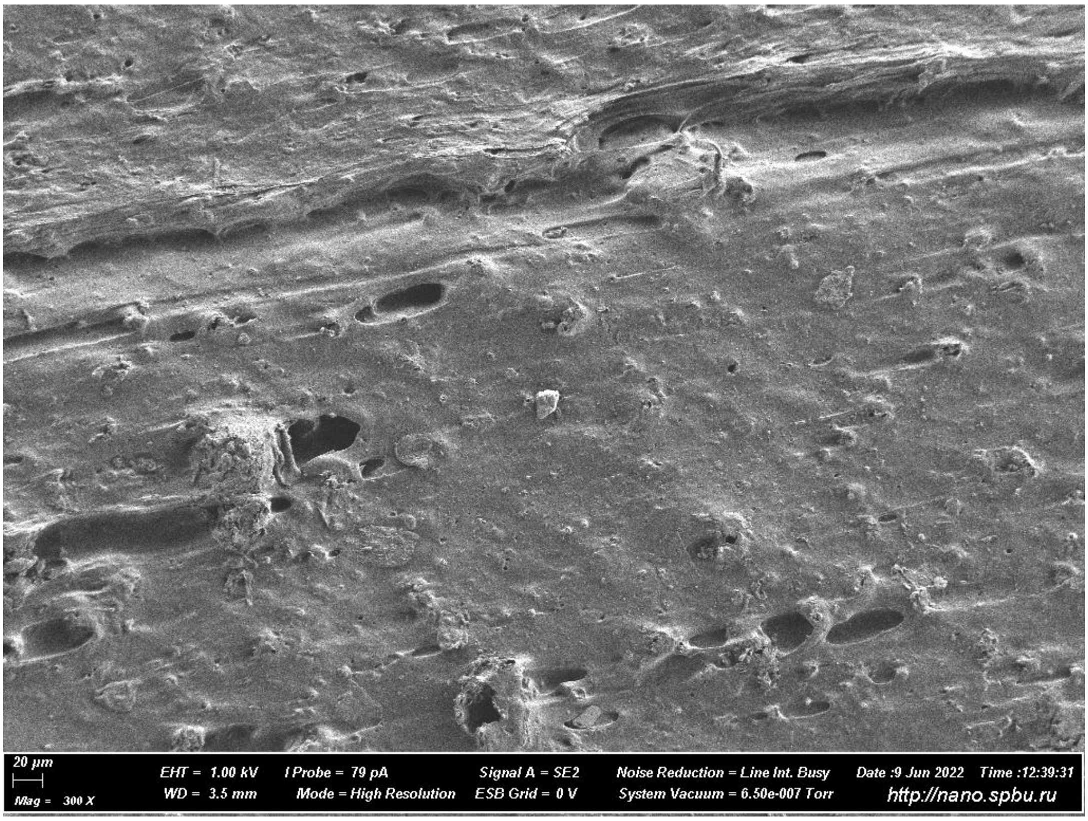

Figure S28. SEM image of the single layer printed with composite (Nylon and 20 mas.% of CCR). Scale bar is 20 µm.

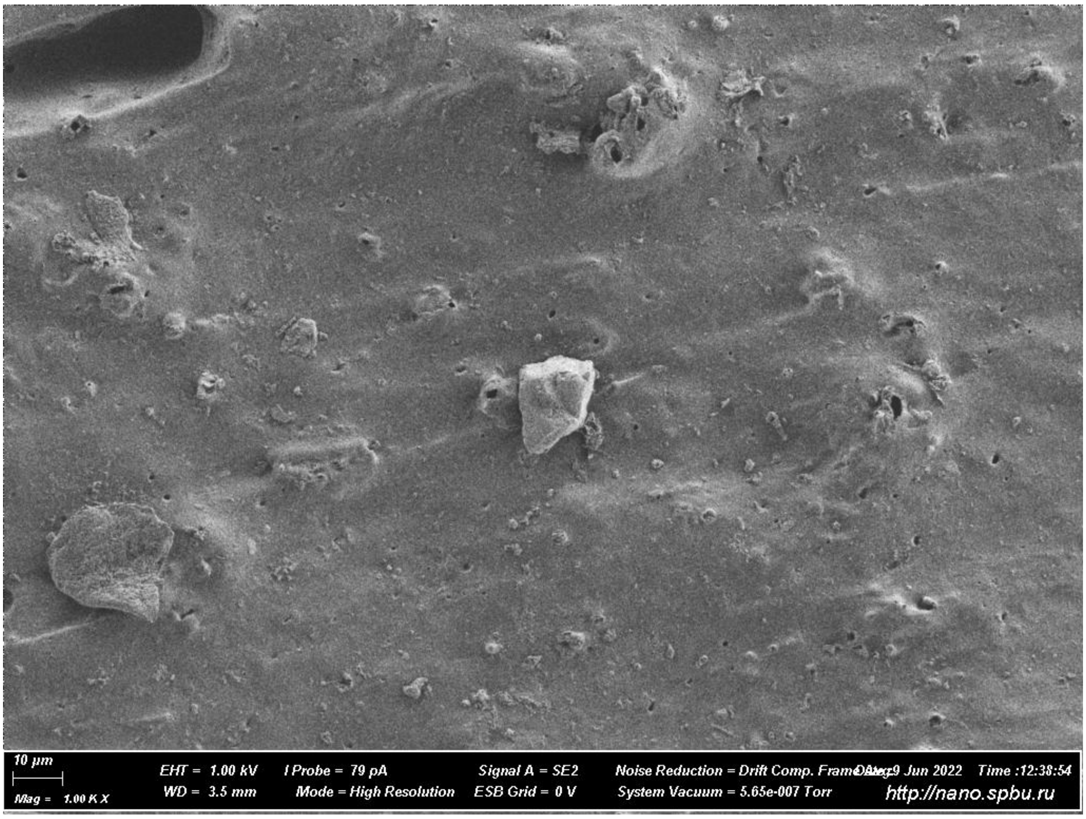

Figure S29. SEM image of the single layer printed with composite (Nylon and 20 mas.% of CCR). Scale bar is 10 µm.

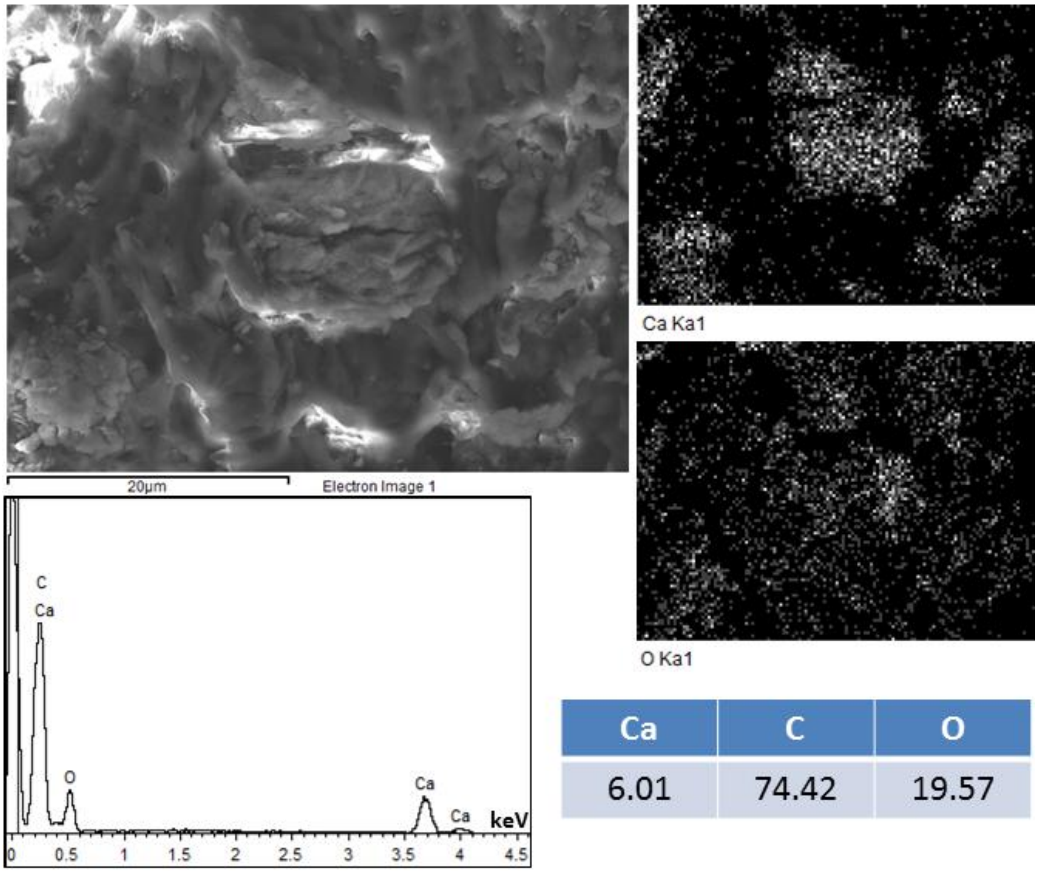

Ca	C	O
6.01	74.42	19.57

Figure S30. EDX map of elements on the Nylon-CCR based composite (in atomic%).

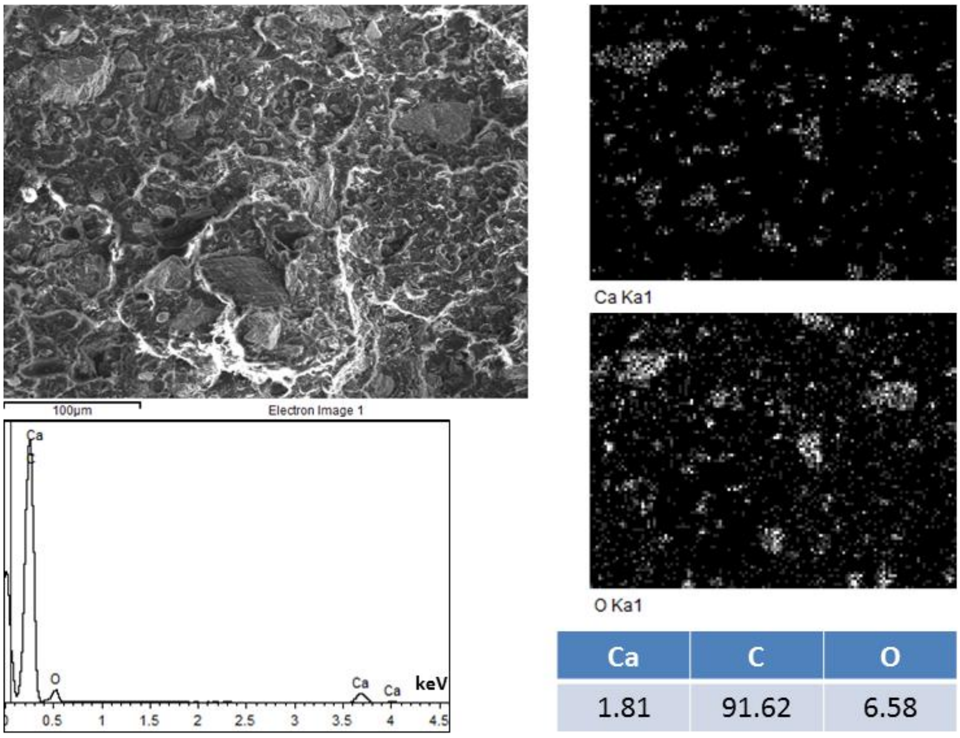

Ca	C	O
1.81	91.62	6.58

Figure S31. EDX map of elements on the HIPS-CCR based composite (in atomic%).

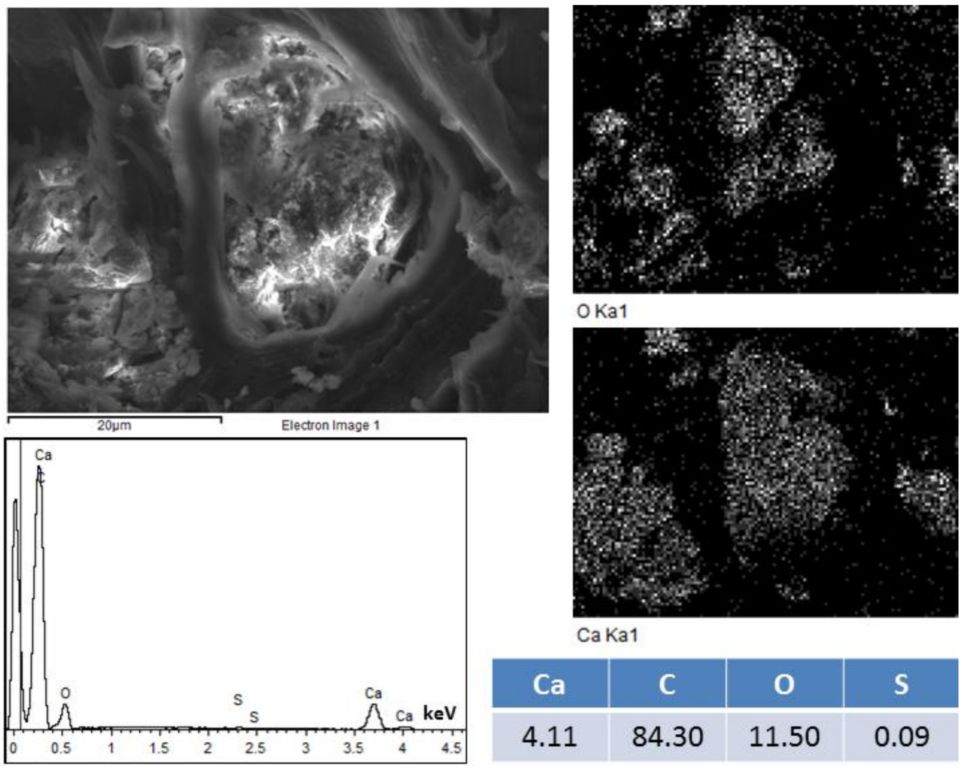

Ca	C	O	S
4.11	84.30	11.50	0.09

Figure S32. EDX map of elements on the SBS-CCR based composite (in atomic%).

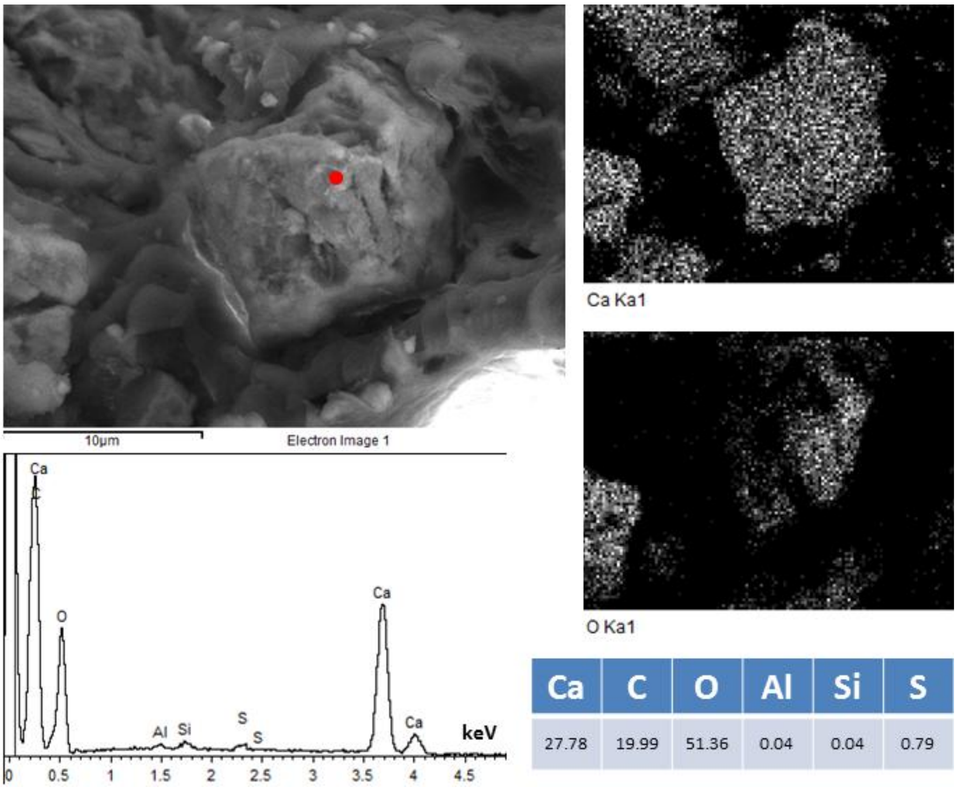

Ca	C	O	Al	Si	S
27.78	19.99	51.36	0.04	0.04	0.79

Figure S33. EDX map of elements on the PETG-CCR-based composite (in atomic%). The red point denotes the selected area for EDX detection.

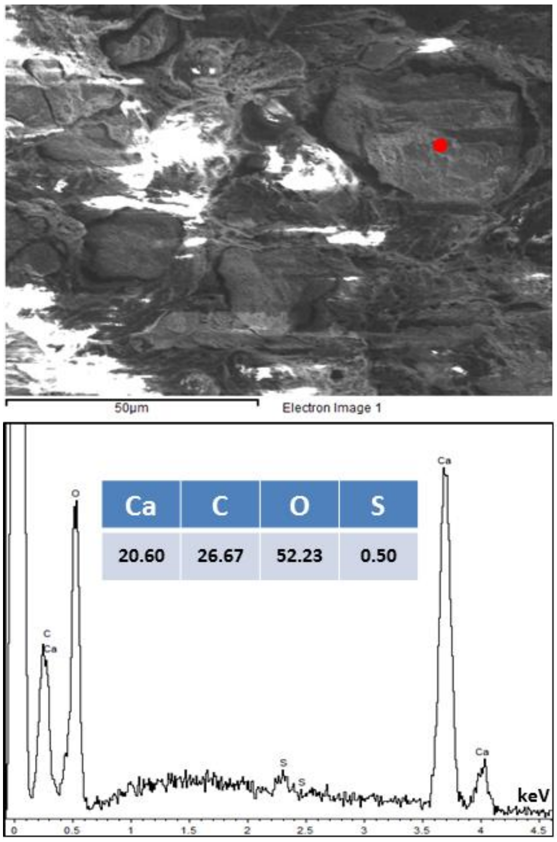

Ca	C	O	S
20.60	26.67	52.23	0.50

Figure S34. EDX map of elements on the ABS-CCR-based composite (in atomic%). The red point denotes the selected area for EDX detection.

S4. 3D printed samples

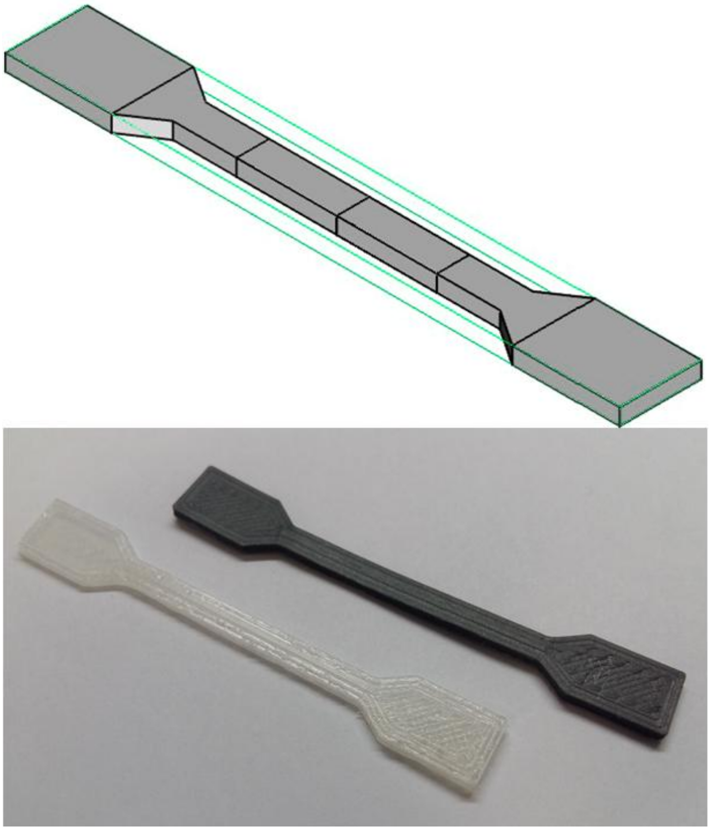

Figure S35. Image of the paddles printed for tensile tests (material – SBS and 20 mas.% of CCR).

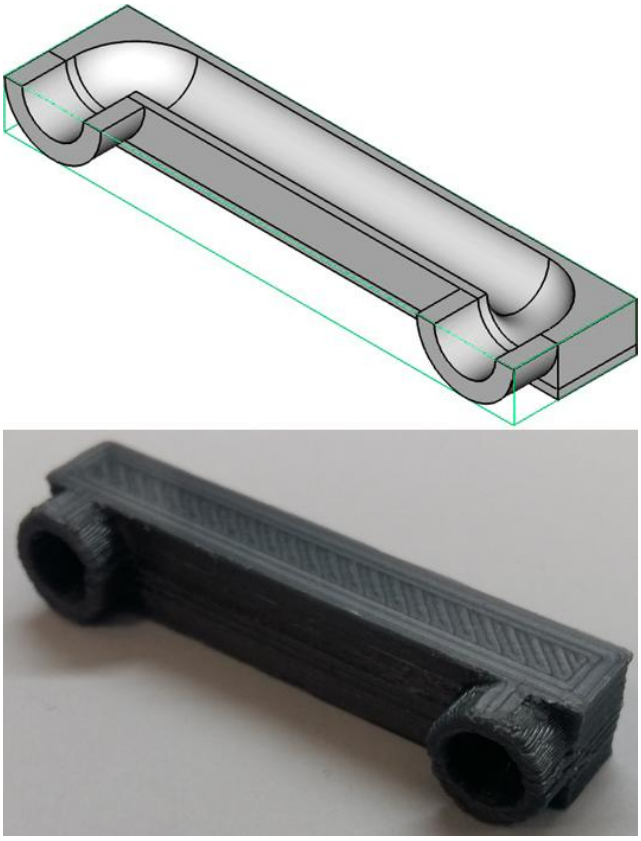

Figure S36. Image of the tube channel for shrinkage test (material – SBS and 20 mas.% of CCR).

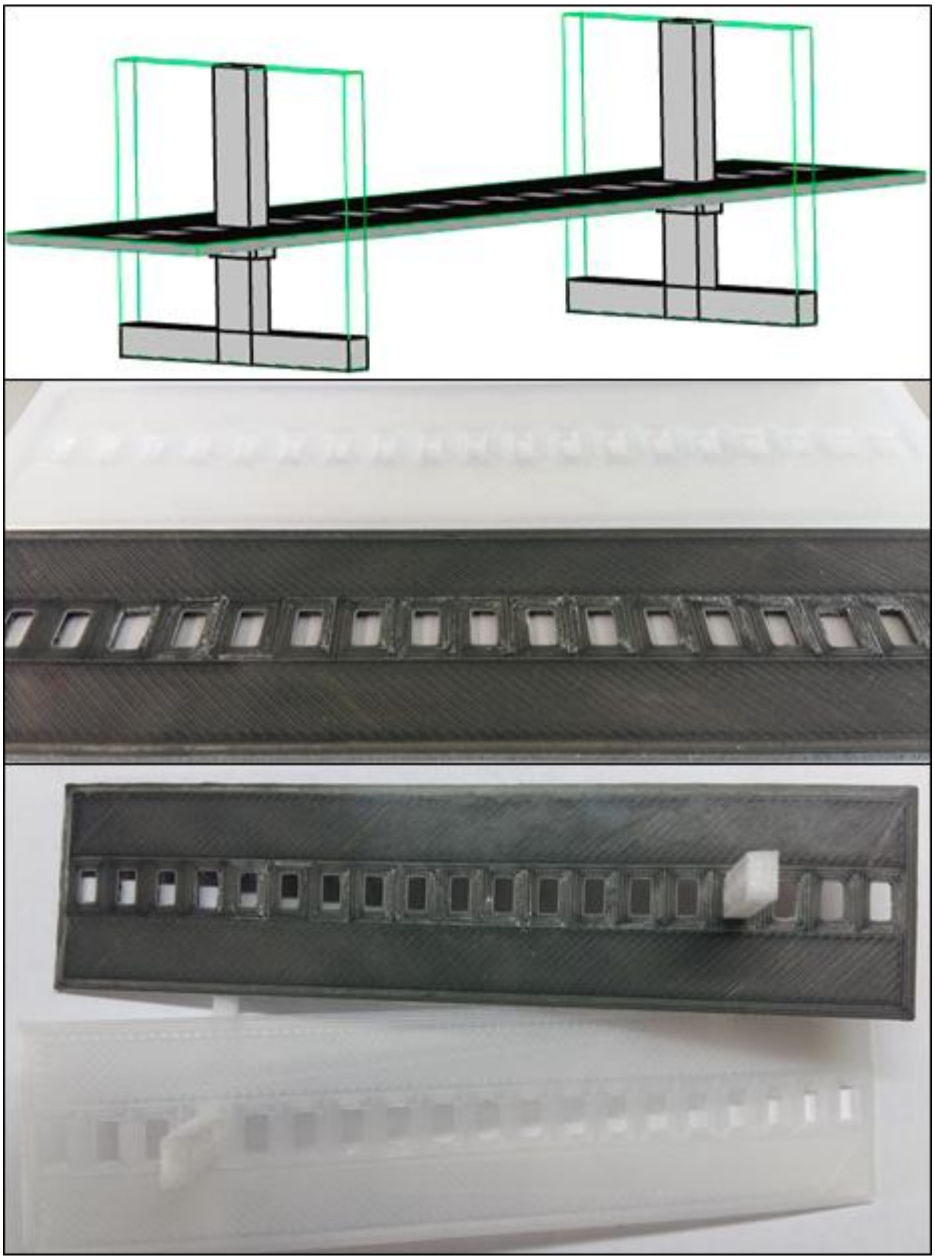

Figure S37. 3D printed bridge with Nylon composite (Nylon and 28 mas.% of CCR).

S5. XRD investigation of CCR obtained

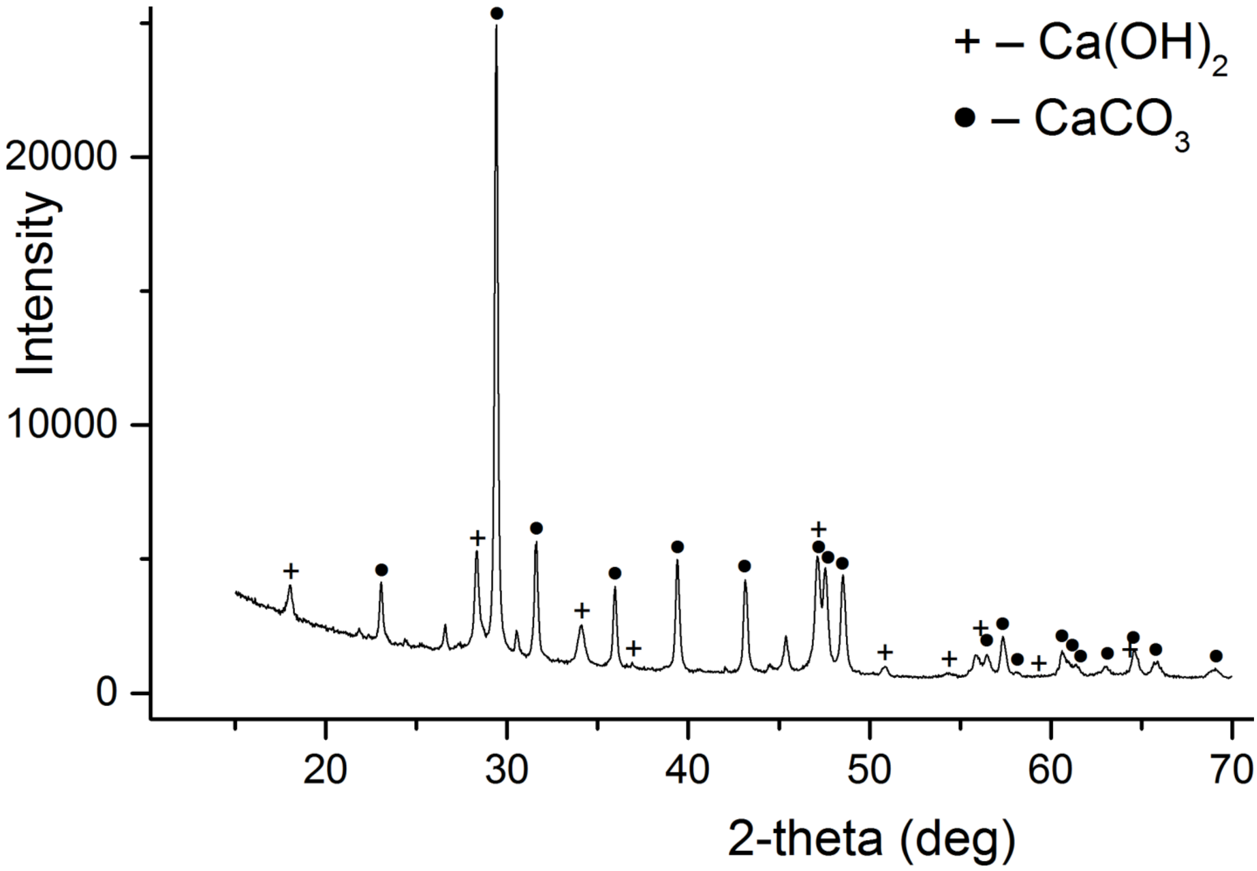

Figure S38. XRD of CCR after drying in oven.

S6. Quality tests of the printed samples

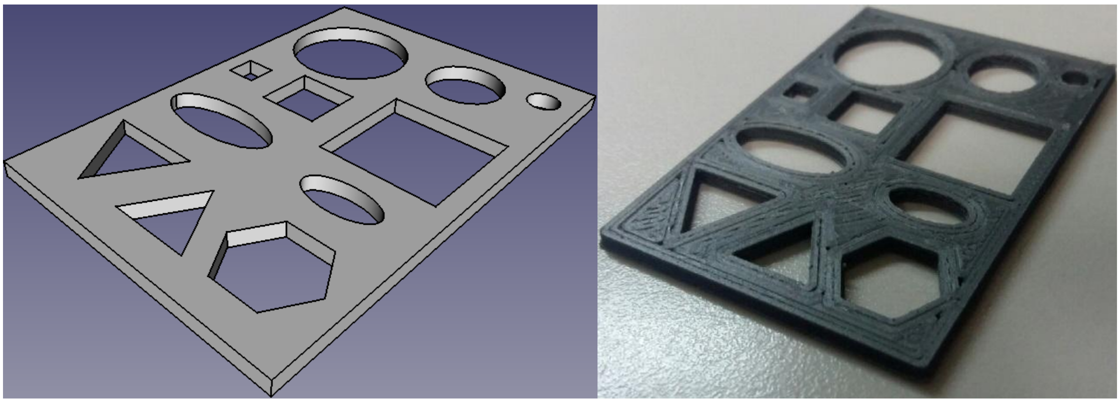

Figure S39. 3D model and printed sample for quality testing.

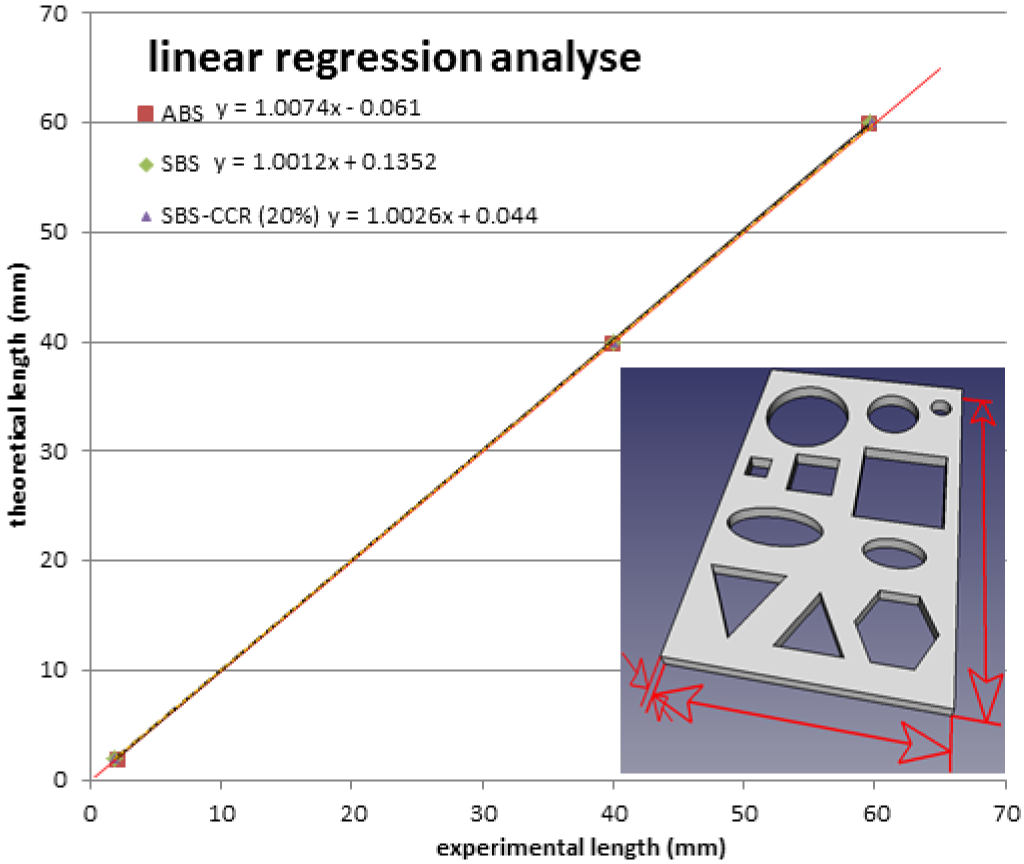

Figure S40. Dimensional changes from digital model to printed sample; linear dimensions were taken for analysis. The red line is the theoretical target slope.

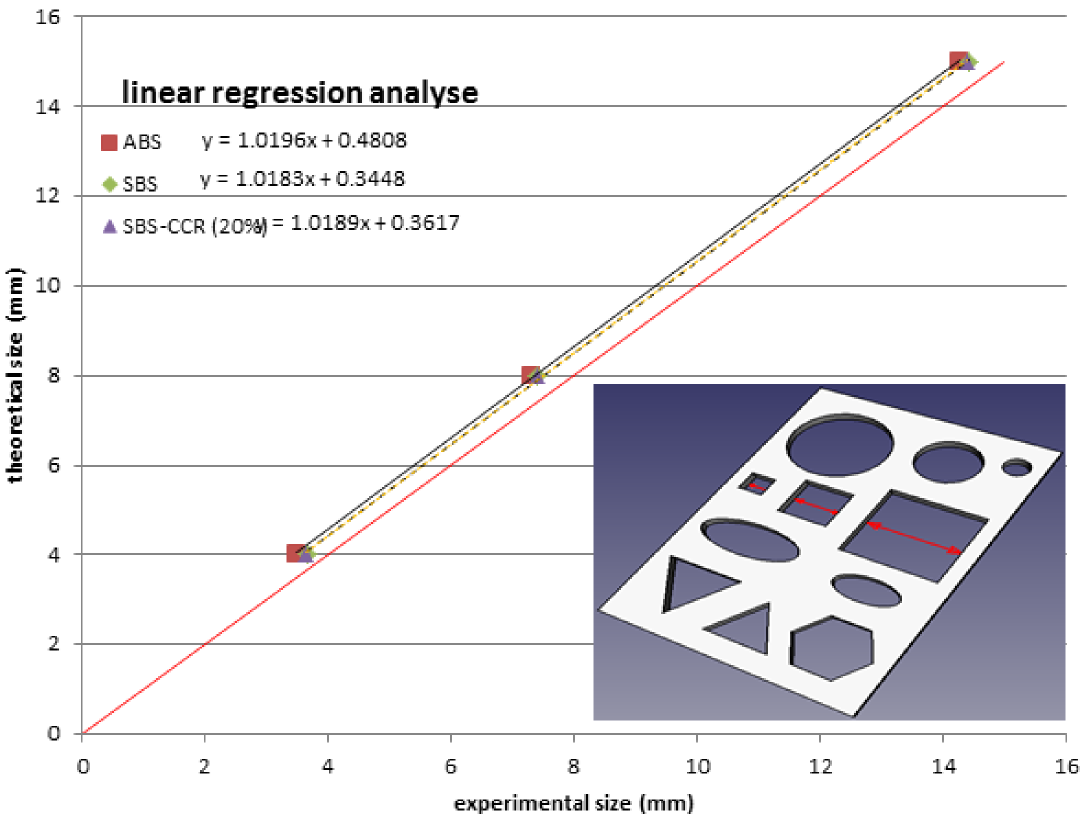

Figure S41. Dimensional changes from digital model to printed sample; square size were taken for analysis. The red line is the theoretical target slope.

The quality of the print was evaluated by comparing different sizes of the printed part with the sizes specified in the original 3D model. ABS was chosen as the hard plastic for printing quality comparison, while SBS was the soft plastic. The composite material – SBS with 20 mas.% of CCR – served as a control sample. According to the created 3D model, the overall dimensions (length, width, and height) and volume are 6 mm, 4 mm, 2 mm, and 3.1 cm^3. The average volume values for the printed samples of ABS, SBS, and SBS-CCR were 3.1 cm^3, 2.6 cm^3, and 2.9 cm^3, respectively. Linear regression analysis showed how the dimensions of the printed part change relative to the modeled object. As a result, we learned that commercially available plastics and the composite material used for printing have little difference in the printed product relative to the theoretical model. In the comparison of linear dimensions (Fig. S40), there is almost no difference with the theoretical dimensions since the linear regression functions practically coincide with the theoretical function. In the case of the dimensions of the internal parts (Fig. S41), these differences increase for all materials used. Such an effect was expected because of the use of a nozzle with a sufficiently large diameter (0.8 mm). Despite this, it should be noted that the regression lines of the directly used materials practically coincide with each other. All internal cutouts of different geometric shapes were printed without significant differences from each other. This once again demonstrated that the quality of the SBS-CCR is on par with the quality of the original plastics.

S7. Tensile tests

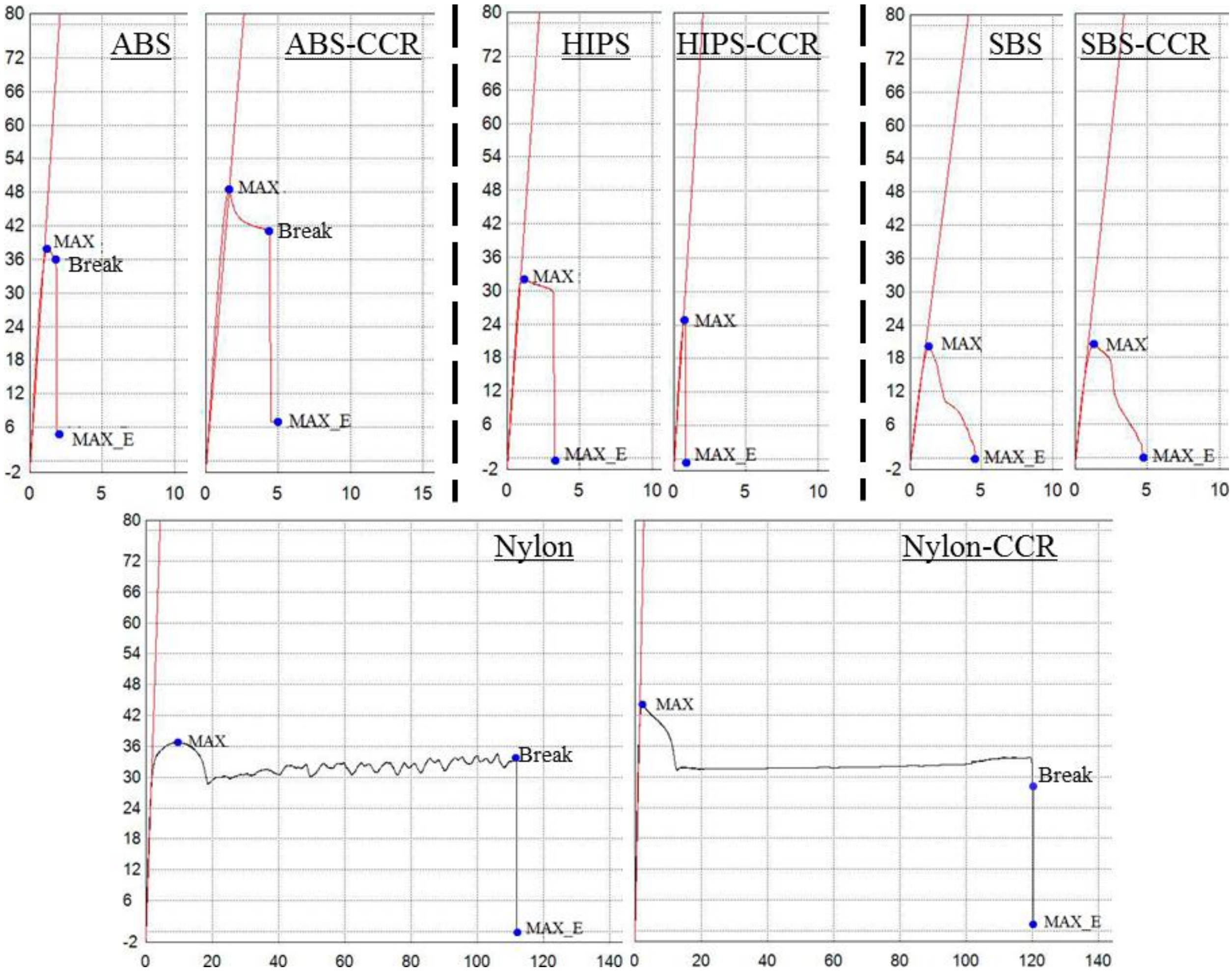

Figure S42. Selected stress-strain curves of the studied composite materials. The left scale is the tensile strength (MPa), and the bottom scale is the elongation of the samples at break (mm). MAX corresponds to the maximum load without sample deformation; MAX_E corresponds to the maximum elongation at break.
